# Cost-Effectiveness of the ‘One4All’ HIV Linkage Intervention in Guangxi Zhuang Autonomous Region, China

**DOI:** 10.1371/journal.pone.0167308

**Published:** 2016-11-28

**Authors:** Xiao Zang, Houlin Tang, Jeong Eun Min, Diane Gu, Julio S. G. Montaner, Zunyou Wu, Bohdan Nosyk

**Affiliations:** 1 British Columbia Centre for Excellence in HIV/AIDS, Vancouver, British Columbia, Canada; 2 Faculty of Health Sciences, Simon Fraser University, Burnaby, British Columbia, Canada; 3 The National Center for AIDS/STD Control and Prevention, Chinese Center for Disease Control and Prevention, Beijing, China; 4 Division of AIDS, Faculty of Medicine, University of British Columbia, Vancouver, British Columbia, Canada; Fudan University, CHINA

## Abstract

**Background:**

In Guangxi Zhuang Autonomous Region, China, an estimated 80% of newly-identified antiretroviral therapy (ART)-eligible patients are not engaged in ART. Delayed ART uptake ultimately translates into high rates of HIV morbidity, mortality, and transmission. To enhance HIV testing receipt and subsequent treatment uptake in Guangxi, the Chinese Center for Disease Control and Prevention (CDC) executed a cluster-randomized trial to assess the effectiveness and cost-effectiveness of a streamlined HIV testing algorithm (the One4All intervention) in 12 county-level hospitals.

**Objective:**

To determine the incremental cost-effectiveness of the One4All intervention delivered at county hospitals in Guangxi, China, compared to the current standard of care (SOC).

**Perspective:**

Health System.

**Time Horizon:**

1-, 5-and 25-years.

**Methods:**

We adapted a dynamic, compartmental HIV transmission model to simulate HIV transmission and progression in Guangxi, China and identify the economic impact and health benefits of implementing the One4All intervention in all Guangxi hospitals. The One4All intervention algorithm entails rapid point-of-care HIV screening, CD4 and viral load testing of individuals presenting for HIV screening, with same-day results and linkage to counselling. We populated the model with data from the One4All trial (CTN-0056), China CDC HIV registry and published reports. Model outcomes were HIV incidence, mortality, costs, quality-adjusted life years (QALYs), and the incremental cost-effectiveness ratio (ICER) of the One4All intervention compared to SOC.

**Results:**

The One4All testing intervention was more costly than SOC (CNY 2,182 vs. CNY 846), but facilitated earlier ART access, resulting in delayed disease progression and mortality. Over a 25-year time horizon, we estimated that introducing One4All in Guangxi would result in 802 averted HIV cases and 1629 averted deaths at an ICER of CNY 11,678 per QALY gained. Sensitivity analysis revealed that One4All remained cost-effective at even minimal levels of effectiveness. Results were robust to changes to a range of parameters characterizing the HIV epidemic over time.

**Conclusions:**

The One4All HIV testing strategy was highly cost-effective by WHO standards, and should be prioritized for widespread implementation in Guangxi, China. Integrating the intervention within a broader combination prevention strategy would enhance the public health response to HIV/AIDS in Guangxi.

## Introduction

Experimental and observational studies across diverse patient populations worldwide have confirmed the individual and public health benefits of antiretroviral therapy (ART) [1,2,3,4,5]. However, in many settings, including China, significant proportions of people living with HIV/AIDS (PLHIV) are lost at each step along the continuum of HIV care, a patient pathway which includes diagnosis, linkage to care, ART engagement, and ultimately viral suppression [6,7].

Strategies to seek, test, treat and retain PLHIV provide a process framework for engaging HIV-positive, treatment-eligible patients in ART with the ultimate goal of helping patients achieve and sustain viral suppression, thereby extending their lives and reducing onward HIV transmission. Engaging and retaining PLHIV in HIV care presents perhaps the greatest challenge to achieving international goals of reducing HIV morbidity, mortality and transmission [8].

In 2011, Guangxi Zhuang Autonomous Region, China, with 46.5 million population, had an estimated HIV prevalence of 80,000–100,000 (10% to 13% of the national total of 780,000) [9]. Only 40% of those who screened HIV-positive at hospital settings received confirmatory testing, and only 85% of those confirmed to be HIV-positive received their HIV test results, resulting in substantial cascade leakage at the point of diagnosis. Furthermore, 38% of PLHIV who were notified of their HIV-positive status failed to receive CD4 testing. As a result, nearly 80% of newly-identified, ART-eligible patients in Guangxi are not engaged in ART [10]. These missed opportunities for patient engagement in HIV care ultimately translate into high rates of developing AIDS and mortality. Guangxi had the highest newly-reported AIDS cases (7,571, 19% of the national total), and the highest number of AIDS-related deaths (3,852, 22% of the national total) among all provinces in China [11].

The CTN-0056 study was designed to address deficits in HIV diagnosis and linkage to care. The ‘One4All’ test intervention entailed a streamlined testing algorithm consisting of rapid point-of-care (POC) HIV testing, POC CD4 testing, and viral load (VL) testing with same-day results and linkage to post-CD4 counselling [12]. SOC, on the contrary, employed multistage stepwise procedures requiring initial screening testing, western blot confirmation testing, traditional CD4 testing and multiple counselling at discrete times prior to treatment initiation, resulting in significant disengagements along each step and typically a ≥15 days delay in initiating ART for those eligible. Piloted in 12 hospitals across Guangxi Zhuang Autonomous Region and implemented by the National Center for AIDS/STD Control and Prevention of the Chinese Center for Disease Control and Prevention (NCAIDS, China CDC), the trial was positioned for immediate scale-up across the region if deemed effective in increasing the adjusted odds of HIV test receipt and subsequent ART uptake among those eligible.

Prioritizing cost-effective interventions is central to developing an effective and sustainable public health strategy for HIV/AIDS. Although a wide variety of interventions have proven effective in improving HIV care along the continuum, and many more currently being tested in a wide range of settings [13], systematic reviews summarizing cost-effectiveness analysis for HIV care interventions noted large knowledge gaps in the literature, particularly on linkage interventions [14,15]. Moreover, many HIV care interventions are executed without formal economic evaluations [16,17,18]. Formal economic evaluation, built upon a dynamic transmission modeling framework, can provide credible evidence of the value of these interventions, thus establishing an evidence-based framework for priority setting and resource allocation.

Our objective was to determine the incremental cost-effectiveness of the One4all testing intervention compared to the current standard of care delivered in county hospitals in Guangxi Zhuang Autonomous Region, China. We populated a previously-validated dynamic HIV transmission model [19,20] with trial data and routinely collected treatment engagement and disease progression data for the region of Guangxi in order to determine the cost-effectiveness of the One4All intervention compared to the standard of care over a 25-year time horizon.

## Methods

### 2.1 The CTN-0056 Trial

CTN-0056 was a cluster-randomized trial conducted simultaneously in 12 county general hospitals which recruited a total of 478 adults who actively sought for inpatient or outpatient care in selected hospitals and screened positive on an initial HIV enzyme immunoassay (EIA) between February 24, 2014 and November 25, 2014 and were followed for 12 months. Study hospitals were selected for homogeneity in structural characteristics, past patient caseloads, and testing procedures. Hospitals were randomized to (1) the One4All test intervention, or (2) the control condition, 6 in each arm, consisting of the current standard of care (SOC), resulting in 232 patients in One4All arm and 246 patients in the SOC arm. There were no significant differences between the arms in patient demographics except that the One4all arm had more participants who completed middle school or higher (40.1%) than SOC (22.8%; p = 0.0498). The trial received approval from the Institute Review Board of the NCAIDS, China CDC, and the University of California, Los Angeles Institutional Review Board. This trial is registered with ClinicalTrials.gov, with identifier number (NCT02084316).

The primary outcome of the trial was defined as the proportion of participants who achieved testing completeness and received their test results and post-test counseling within 30 days, given they have received a positive HIV result on the initial EIA screening. Testing completeness is defined as completion of three required components: (1) Initial EIA; (2) CD4 testing and (3) confirmatory testing–western blot in the SOC, or confirmatory VL in the One4all arm. The secondary outcome is the proportion of ART eligible participants who initiate ART within 90 days of their initial HIV screen positive test. The timing and components of the One4All intervention versus SOC are presented in Table 1.

**Table 1:** Timing and components of the One4All Intervention and Standard of Care (SOC).

	Standard of Care (SOC)	One4All Intervention
Baseline	• 2 point-of-care HIV screening tests + counseling • Western blot confirmatory test blood draw	• 2 point-of-care HIV screening tests + counseling • Point-of-care CD4 count blood draw • CD4 results + counseling • Viral load test blood draw
Follow-up 1 (10–15 days)	• Western blot results + counseling • CD4 count blood draw	• Viral load results + counseling*
Follow-up 2 (10–15 days for SOC)	• CD4 results + counseling*	• ART counseling + initiation
Follow-up 3	• ART counseling + initiation • Viral load test blood draw (after ART initiation)	

*Indicates point of testing completeness

### 2.2 Study Design

We adapted and extended an existing deterministic transmission model previously used to estimate the health benefits and costs of expanded HIV screening and ART in the United States [19], British Columbia, Canada [20], and China [21]. Our data sources included results of the CTN-0056 trial, primary analysis of the China National HIV/AIDS Comprehensive Response Information Management System (CRIMS) and published epidemiological and behavioural data to estimate HIV prevalence, incidence, quality-adjusted life-years (QALYs), health care costs and incremental cost-effectiveness ratios (ICERs) associated with the One4All intervention, compared to the standard of care in Guangxi Zhuang Autonomous Region, China, from 2014–2038. A 25-year time horizon was chosen in order to capture potential second-order HIV preventative benefits, however results were reported at 1- and 5-year time horizons as well.

**Fig 1:**
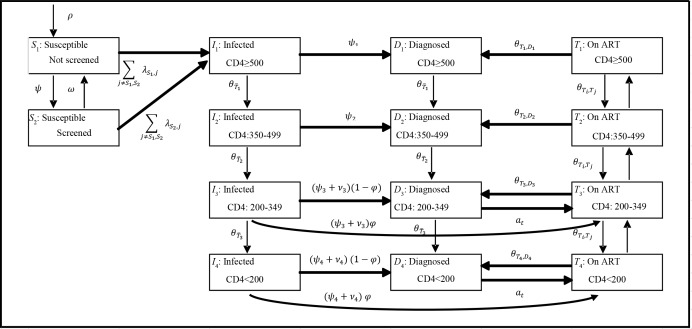
Model Diagram.

A schematic of the model and dynamics is presented in Fig 1. The adult population of Guangxi aged 15–64 were partitioned into compartments on the basis of HIV risk behavior (men who have sex with men (MSM), injection drug users (IDU), MSM/IDU, and heterosexual (HETERO)), screening status (screened in the past 12 months or not) and HIV infection status. Among those HIV-infected, individuals were further classified as infected, diagnosed, and on ART, and partitioned according to CD4 cell count (CD4≥500 cells/mm^3^, 350–499, 200–349, <200). Health state transitions occurred at monthly intervals. It is important to note that our model captures individuals that are both infected and never diagnosed (the second ‘column’ of compartments in Fig 1), and diagnosed but not engaged in treatment (third column of compartments). We further note that the population of diagnosed patients is dynamic and composed, at any given time, of ART-experienced and ART-naïve PLHIV.

We simulated the HIV epidemic by first instantiating the risk group size and HIV prevalence levels based on Guangxi data in 2010, and population figures from the Guangxi Statistical Yearbook and 2010 Guangxi census [10,22], thus using 2011–2014 as an instantiation period for the model. We calibrated our model to replicate observed annual diagnoses by adjusting the ratio of monthly screening rate for high (IDU, MSM and IDU/MSM) versus low (HETERO) risk group in compartment i ($\psi_{i}^{h}/\psi_{i}^{l}$). We also calibrated our model to match the observed number of PLHIV on ART in Guangxi by adjusting the rate at which an individual in diagnosed compartment i entered treatment at a given CD4-based state of disease progression at time t (*φ*,*α*_*it*_).

Following calibration to match the known annual number of HIV diagnoses and total number of PLHIV on ART, the model was validated to ensure key epidemiological parameters approximated known or externally-estimated figures. We focused on the size of the HIV-negative population (aged 15–64), the total number of diagnosed cases, and the annual number of deaths among diagnosed PLHIV.

Cost-effectiveness analysis was conducted in accordance with well-established guidelines on cost-effectiveness analyses and dynamic transmission modeling [23]. We considered a public health system perspective. All costs were presented in 2014 Chinese Yuan Renminbi (Chinese currency, USD1 = CNY 6.20 in year 2014), and discounted costs and QALYs at an annual rate of 3%. Key parameters are presented in Table 2, with further detail regarding model construction, parameterization, calibration and validation provided in S1 Appendix.

**Table 2:** Model parameter estimates.

Variable	Values	References
***Population Demographics***		
Initial population (aged 15–64):	31,832,050	[22]
IDU	293,551	[24]
MSM	326,797	[25]
MSM/IDU	28,417	[26]
HETERO	31,183,285	Calculated
Initial HIV prevalence (%):	97,114	[9]
IDU	27,349	[27]
MSM	12,418	[28,29]
MSM/IDU	2,648	[28,29]
HETERO	54,699	[30]
Initial no. of HIV diagnosed (including on ART):	50,001	Guangxi CDC
IDU	14,566	Guangxi CDC
MSM	4,595	Guangxi CDC
MSM/IDU	980	Guangxi CDC
HETERO	29,861	Guangxi CDC
Initial no. on ART:	14,749	Guangxi CDC
IDU	2,563	Guangxi CDC
MSM	315	Guangxi CDC
MSM/IDU	25	Guangxi CDC
HETERO	11,846	Guangxi CDC
Monthly mortality rate for individuals:		
Monthly mortality rate (background) (aged 15–64 years)		
HETERO or MSM	0.00021	[10,22]
IDU or MSM/IDU	0.00232	[21]
Monthly mortality rate (background) (aged 65–95 years)	0.00326	[10,22]
Monthly mortality rate (HIV infected and diagnosed)		
HETERO or MSM		
Asymptomatic (CD4: ≥350)	0.00225	Calculated, [21]
Symptomatic (CD4: 200–349)	0.00600	Calculated, [21]
AIDS (CD4: <200)	0.02120	Calculated, [21]
IDU or MSM/IDU		
Asymptomatic (CD4: ≥350)	0.00441	Calculated, [21]
Symptomatic (CD4: 200–349)	0.00826	Calculated, [21]
AIDS (CD4: <200)	0.02387	Calculated, [21]
Monthly mortality rate (on ART)		
HETERO or MSM		
CD4: ≥500	0.00049	Appendix
CD4: 350–499	0.00052	Appendix
CD4: 200–349	0.00063	Appendix
CD4: <200	0.00268	Appendix
IDU or MSM/IDU		
CD4: ≥500	0.00261	Calculated
CD4: 350–499	0.00264	Calculated
CD4: 200–349	0.00275	Calculated
CD4: <200	0.00485	Calculated
Monthly maturation rate	0.00058	[10,22]
Monthly entry rate (background)	0.00179	[10,22]
***Sexual Transmission***		
Transmission probability per partnership:		
Heterosexual:		
CD4: ≥500	0.030	[21,31,32,33,34,35,36]
CD4: 350–499	0.030	[21,31,32,33,34,35,36]
CD4: 200–349	0.040	[21,31,32,33,34,35,36]
CD4: <200	0.080	[21,31,32,33,34,35,36]
Homosexual:		
CD4: ≥500	0.040	[21,37,38,39,40]
CD4: 350–499	0.050	[21,37,38,39,40]
CD4: 200–349	0.050	[21,37,38,39,40]
CD4: <200	0.100	[21,37,38,39,40]
Annual same-sex partners (N)	5	[41]
Annual opposite-sex partners (N):		
IDU	3	[42]
MSM	0.3	[43]
MSM/IDU	0.3	[43]
HETERO	1.2	[30]
Condom use with same-sex partners:		
MSM	46.40%	[27]
MSM/IDU	46.40%	[27]
Condom use with opposite-sex partners:		
IDU	38.30%	[44]
MSM	29.50%	[45]
MSM/IDU	29.50%	[44,45]
HETERO	11.00%	[41]
Condom effectiveness	0.80	[46,47,48]
***Injection Drug Use Transmission***		
Transmission probability per shared injection:		
CD4: ≥500	0.002	[20,21,49,50]
CD4: 350–499	0.002	[20,21,49,50]
CD4: 200–349	0.003	[20,21,49,50]
CD4: <200	0.003	[20,21,49,50]
Average injections per year	200	[19,21,42]
Fraction of injections that are shared, %	26.40%	[27]
***HIV Screening***		
Monthly HIV screening rate:		
High risk (IDU or MSM)	Time-varying	Calibrated
Low risk (HETERO)	Time-varying	Calculated, [51]
One4All intervention screening rate multiplier	3.45	One4All, [52]
Monthly probability of symptom-based case-finding:		
High risk (CD4: <200)	0.00923	[21,53]
Low risk (CD4: 200–349)	0.02082	[21,53]
Reduction in partner numbers among diagnosed (HIV)	0.20	[21,54]
Reduction in partner numbers among diagnosed (AIDS)	0.90	[21]
***Antiretroviral therapy***		
Rate of ART entry from diagnosed compartments	Time-varying	Calibrated
Fraction of individuals from infectious to start ART	0.3	[21,55]
Reduction in injection infectivity due to ART	0.50	[19,53]
Reduction in sexual infectivity due to ART:		
Same sex	0.90	[21,37,38,39,40]
Opposite sex	0.90	[21,37,38,39,40]
***HIV disease progression rates for individuals not on ART***		
CD4≥500 to CD4: 350–499	0.02209	[20,53,56,57,58]
CD4: 350–499 to CD4: 200–349	0.02209	[20,53,56,57,58]
CD4: 200–349 to CD4: <200	0.02209	[20,53,56,57,58]
CD4: <200 to Death	0.00250	[20,53,56,57,58]
***HIV disease progression rates for individuals on ART***	Time-varying	Appendix
***Health-Related Quality of Life***		
Susceptible	0.08333	
Infected: CD4: ≥500	0.07500	[21,53,59,60,61,62]
Infected: CD4: 350–499	0.06583	[21,53,59,60,61,62]
Infected: CD4: 200–349	0.06583	[21,53,59,60,61,62]
Infected: CD4: <200	0.06000	[21,53,59,60,61,62]
Diagnosed: CD4: ≥500	0.07083	[21,53,59,60,61,62]
Diagnosed: CD4: 350–499	0.06000	[21,53,59,60,61,62]
Diagnosed: CD4: 200–349	0.06000	[21,53,59,60,61,62]
Diagnosed: CD4: <200	0.05667	[21,53,59,60,61,62]
On ART: CD4: ≥500	0.07083	[21,53,59,60,61,62]
On ART: CD4: 350–499	0.06917	[21,53,59,60,61,62]
On ART: CD4: 200–349	0.06917	[21,53,59,60,61,62]
On ART: CD4: <200	0.06833	[21,53,59,60,61,62]
IDU multiplier	0.90	[20,63,64]
***Costs (2014 CNY)***		
Monthly non-ART healthcare cost for non-IDU:		
CD4: ≥350	2,029	[21,65]
CD4: 200–349 untreated	3,350	[21,65]
CD4: 200–349 treated with ART	2,996	[21,65]
CD4: <200 untreated	4,491	[21,65]
CD4: <200 treated with ART	3,916	[21,65]
Monthly non-ART healthcare cost for IDU:		
CD4: ≥350	4,830	[20,66]
CD4: 200–349 untreated	7,974	[20,66]
CD4: 200–349 treated with ART	7,131	[20,66]
CD4: <200 untreated	10,689	[20,66]
CD4: <200 treated with ART	9321	[20,66]
Monthly cost of ART	221	Guangxi CDC
Monthly healthcare costs for HIV negatives:		
IDU or MSM/IDU	196	[20,21,66]
MSM	78	[20,21,66]
HETERO	82	[20,21,66]
Cost of HIV ELISA antibody test	24	Guangxi CDC
Cost of POC* HIV screening test	24	Guangxi CDC
Cost of confirmatory western blot test	500	Guangxi CDC
Cost of behavior counselling	82	[21,67]
Cost of CD4 count test	240	[21,67]
Cost of POC* CD4 count test	576	[68,69]
Cost of plasma viral load test	1500	Guangxi CDC
Annual discount rate, %	3	[21]

* POC represents ‘point-of-care’ in the proposed One4All screening intervention

### 2.3 Integrating CTN-0056 trial results

The primary outcome of the trial was integrated into the model through the parameter ψ (Fig 1), capturing the increase in the adjusted odds of test receipt within one month in the One4All cohort, compared to the standard of care. The effect of the intervention was adjusted for individual and hospital-level effects using a generalized linear mixed effects regression framework (log link, beta distribution, controlling for hospital-level random effects) [70], and was implemented among HIV-infected heterosexuals with CD4<350, mirroring the study population. This effect (odds ratio of 21.78) was converted to a relative risk (3.45) and entered multiplicatively with the base HIV testing rate model parameter, following standard practice [52]. The effect of the intervention was implemented as incremental to the base-case, or standard of care, which was the calibrated representation of the Guangxi HIV/AIDS epidemic at the time of trial initiation (2014). The costs of the One4All intervention were higher than the current standard of care (CNY 2,182 vs. CNY 846), accounting for the higher costs of POC CD4 testing and an additional viral load test.

### 2.4 The force of HIV infection

We incorporated HIV transmission through heterosexual sexual contact, homosexual sexual contact and needle sharing associated with injection drug use. Heterosexual sexual contact occurs within risk groups (for example, both partners are heterosexual, both are injection drug users) and across groups (for example, a (female) injection drug user with a (male) heterosexual, or a (female) heterosexual with an MSM partner). We assumed proportional mixing, in which individuals could be infected by those of other risk groups, but the probability of infection was proportional to the level of contact (sexual or injection) between groups. Parameters dictating HIV risk behaviors were allowed to change over time according to proxies of injection and sexual risk behavior–specifically, rates of methadone maintenance treatment (MMT) uptake and non-HIV sexually transmitted disease [71] during the study period. The model also accounts for changes in risky behavior due to effective HIV screening and counseling [54].

### 2.5 Disease progression

Disease progression was differentiated among those on ART and not on ART, and estimated as a function of CD4 count, stratified into the four categories noted above. We derived monthly transition rates from population-level registry data held by the NCAIDS, China CDC using a multivariate multi-state Markov model [72]. The methodology allowed for transitions out of treatment, in addition to CD4 improvement and deterioration in treatment (as indicated by arrows in Fig 1). Otherwise, individuals progressed according to the natural history of HIV, for which transition rates were drawn from the published literature [56].

### 2.6 Costs and quality adjusted life years

Healthcare costs considered in this study included costs of delivering HIV testing in the two comparative arms, costs for ART regimen and non-ART medical care among the entire population. The costs of delivering the ‘standard of care’ and ‘One4All’ trial interventions were estimated from trial data. Otherwise, ART and non-ART medical care among PLHIV were estimated from published sources [21]. We applied QALY weights derived from the peer-reviewed literature for HIV-negative individuals and PLHIV in and out of treatment, adjusting for injection drug use [59,60,61,62,73] (further details in S1 Appendix).

### 2.7 Sensitivity analysis

We considered alternative scenarios to test structural and parameter uncertainties within the model (pertaining to movement in the model, and the point estimates of parameters dictating these movements, respectively), as well as the sensitivity of the results to the scale and effectiveness of the trial. First, alternate scenarios were constructed to determine the sensitivity of results to parameters estimated with the greatest level of uncertainty, including the proportion of MSM among susceptible population, number of IDU among susceptible population, and baseline HIV prevalence, respectively. Alternate values for key parameter estimates were drawn from the peer-reviewed literature representing alternate estimates, or national (as opposed to Guangxi Regional) estimates. Second, model results were estimated at both the upper and lower bound of CTN-0056 trial’s primary outcome, the adjusted odds ratio of screening completeness. Third, alternate fitted curves were utilized to alter baseline constant prediction for time-varying parameters. Forth, we considered alternate scenarios whereby the intervention was able to reach a broader population of PLHIV, including MSM, IDU and MSM/IDU whose CD4 counts were below 350 cells/mm^3^. Finally, we executed a threshold sensitivity analysis, varying the primary outcome of the trial within the feasible range of odds ratios (1,∞) on the adjusted measure of effect estimated in the trial. Intuitively, this provides us with information on the thresholds at which the One4All intervention would be deemed cost-inefficient relative to the current standard of care. A full description of all sensitivity scenarios is presented in Table A9 in S1 Appendix.

### 2.8 Ethics Statement

This modeling study has received institutional ethical approval from the University of British Columbia (UBC-PHC approval number: H14-02140).

## Results

The CTN-0056 trial recruited a patient population consisting of 97% heterosexuals, 75% of which were male, and 75% over the age of 45. Among those receiving a CD4 test, median CD4 cell counts were 122 and 143.5 for One4All and SOC, respectively [12]. As such, our baseline comparison introduced the effect of the One4All intervention in only HIV-infected heterosexuals with CD4 count<350.

The full list of parameters used in the model is presented in Table 2. The analysis was parameterized using data from a number of sources, including disease registry data from the NCAIDS, China CDC, the One4All trial, and the published literature. The primary outcome of the trial, the adjusted odds of completeness of HIV screening within 30 days, estimated to be 21.78 (95% CI: 3.81, 124.46), was converted to relative risk 3.45 (95% CI: 2.22, 3.82) and incorporated into the model directly.

The model was calibrated to match the number of new diagnoses, and the number of PLHIV accessing antiretroviral therapy (ART) in a given calendar year between 2011 and 2014, and produced valid estimates of all-cause deaths among PLHIV, and the estimated size of the susceptible population during the calibration period (results presented in S1 Appendix).

If expanded to the entire region of Guangxi, we estimate the One4All intervention would lead to a reduction of 39 incident HIV cases in the first year, and a total of 802 cases, 0.5% of the expected 171,923 incident cases in the standard of care (SOC) scenario over the 25-year study period. Further, we estimated decreases of 45 deaths in the first year, and 1629 deaths over the 25-year time horizon, translating into a decrease of 1.6% in the probability of mortality among PLHIV in Guangxi over the 25-year study period (Table 3).

**Table 3:** Results of cost-effectiveness analysis.

	Incident cases	Deaths among PLHIV	ART costs	Non-ART costs	Total costs	QALYs	ICER
			2014 Million CNY	2014 Million CNY	2014 Million CNY	Million	2014 CNY
***1-year time horizon***							
Standard of care	5,944	5,285	102.46	4,921.04	37,682.24	32.64	
One4All*	5,905	5,240	103.50	4,923.54	37,685.79	32.64	259,978
One4All_All*	5,806	5,203	104.33	4,921.86	37,685.00	32.64	91,970
***5-year time horizon***							
Standard of care	28,184	20,582	577.91	22,705.76	179,643.60	156.27	
One4All*	27,885	20,018	589.83	22,684.93	179,635.39	156.27	Dominant
One4All_All*	27,515	19,823	593.93	22,652.18	179,607.98	156.27	Dominant
***25-year time horizon***							
Standard of care	171,923	103,519	2889.32	99,970.77	738,246.57	635.18	
One4All*	171,121	101,890	2940.29	100,122.22	738,459.09	635.20	11,678
One4All_All*	169,578	101,075	2948.51	99,930.02	738,291.06	635.21	1,690

* *One4All* represents intervention scenario whereby One4All screening intervention was only applied to heterosexual PLHIV, corresponding to trial study population. *One4All_All* represents sensitivity scenario whereby One4All intervention was expanded to all PLHIV from all risk groups.

Within a 5-year time horizon the One4All intervention was a dominant strategy. The additional costs of ART (an increment of CNY 6.2 million) were offset by savings of CNY 17.5 million in non-ART medical costs, which incorporated the higher costs of the One4All screening algorithm. With an incremental gain of 409 QALYs, accumulating primarily via delayed mortality, One4All was a dominant strategy (ie. lower costs, higher QALYs gained). In a 25-year time horizon, as a result of the increased duration of life among those diagnosed, both ART (an increment of CNY 51.0 million) and non-ART medical costs (an increment of CNY 136.5 million) were greater in the One4All strategy. With an estimated gain of 18,199 QALYs, we estimated an ICER of 11,678 CNY per QALYs gained. With a current GDP per capita of CNY 49,351 [74], the One4All strategy is highly cost-effective, according to the World Health Organization (WHO) standards [75] (Table 3).

**Fig 2:**
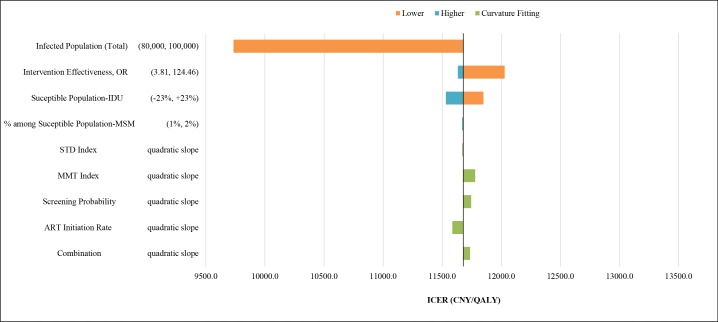
One-way Sensitivity Analyses Plot—Tornado Diagram Estimating Sensitivity of Baseline Results to Uncertain Parameters. Baseline model: (1) 97114 total PLHIV at baseline; (2) Intervention effectiveness, OR = 21.78; (3) 293551 susceptible IDUs; (4) MSM: 1.116% among susceptible population; (5) Constant estimates as year 2014 for STD index, MMT index, annual screening probability and annual ART initiation rate since year 2015.

We executed a series of one-way sensitivity analyses focusing on parameters with the greatest degree of uncertainty to determine their effect on the estimated ICER over a 25-year time horizon. Given the fairly modest epidemiological impact of One4All, and the fact that these changes were applied to both strategies, the ICER varied little across sensitivity analyses. Using the lower bound of the UNAIDS estimate for HIV/AIDS prevalence in Guangxi (80,000, as opposed to the baseline prevalence of 97,000) resulted in the most substantial change amongst all sensitivity scenarios, with an ICER of 9,734 CNY per QALY gained, while the highest ICER, screening intervention effectiveness at lower bound, was only slightly higher than 12,000 CNY/QALY, well within a ‘highly cost-effective’ range. (Fig 2)

If the One4All intervention could reach all risk groups, it could result in a 1.36% reduction in HIV incidence and a 2.36% reduction in mortality among PLHIV, for a cost of 1690 CNY per QALY gained over a 25-year timeframe (Table 3).

Finally, we found that One4All remained cost-effective and highly cost-effective at even minimal levels of effectiveness (odds ratio 1.01 and 1.025, respectively) in HIV testing uptake, further supporting our results.

## Discussion

Using a validated dynamic HIV transmission model synthesizing a wealth of data characterizing the HIV/AIDS epidemic in Guangxi, China, we found the One4All HIV testing intervention to be a highly cost-effective strategy to increase HIV test receipt and subsequent ART engagement. Projected over a 25-year timeframe, if the intervention was to be expanded across the province, we estimated One4All would result in declines of 0.5% and 1.6% in HIV incidence and mortality among PLHIV, respectively, increasing to 1.36% and 2.36% if the intervention could extend to populations of MSM and IDU. Sensitivity analyses supported the robustness of the results, and demonstrated that One4All is likely to remain cost-effective in practice even if the real-world odds or HIV test receipt are lower than observed in the trial.

While the One4All intervention undoubtedly represents a much-needed enhancement to Guangxi HIV testing and ART engagement protocols, if implemented, the estimated impact on the HIV epidemic is expected to be modest, with minimal influence on HIV transmission. This should come as little surprise, as it remains a ‘passive’ intervention, in that undiagnosed cases are not actively sought out for testing. Thus, while optimizing mechanisms to ‘test and treat’, the intervention does not actively seek out undiagnosed cases, and downstream ART retention is not addressed. Furthermore, the trial recruited primarily older male heterosexuals at late stages of disease progression. While reductions in morbidity and mortality are certainly possible in such populations, declines in HIV transmission are likely to be limited, as projected by our model.

Expanding the scope of the intervention to reach individuals at earlier stages of disease progression would entail integrating the testing algorithm into outpatient and preventative care settings. For instance, the very low number of IDU recruited into CTN-0056 suggests that people who inject drugs do not access care through the typical pathways in Guangxi. Using the One4All algorithm, HIV testing could be delivered in methadone maintenance treatment facilities, similar to other settings internationally [76,77]. Otherwise, home, community-based and mobile testing units, among other modes of outreach-oriented testing campaigns have proven cost-effective in settings with moderate and high undiagnosed HIV prevalence [19,78,79].

The One4All intervention should be considered an integral component of a comprehensive HIV prevention and treatment strategy that would also include more active modes of HIV testing to reach undiagnosed populations at earlier stages of disease progression, and in marginalized or hard-to-reach populations, as well as efforts to optimize ART engagement and retention. The concept of combination implementation [80,81,82,83,84] has been introduced to capture the need for pragmatic, evidence-based prevention strategies in real-world settings. An economic modeling framework can inform public health investment in the optimal mix of strategies that provide the greatest health benefits, both across regions and over time [85].

This analysis was not without limitations. First, similar to other models used in cost-effectiveness analyses of HIV screening and treatment [86], infectivity was modeled indirectly through CD4-based disease progression stages. Second, while the probability of transmission in early HIV infection was not modeled explicitly, its long-term impact on reduction of incidence was determined to be minimal [87], and is not likely to influence the intervention under study. Third, while the total susceptible population in Guangxi was reproduced accurately, in- and out-migration of PLHIV is not observed, and was implicitly assumed to be equal to zero. Fourth, while drug resistance was not explicitly modeled in our study, it was accounted for in that the cost and disease progression estimates were derived from statistical analyses and published sources that captured the full population of Guangxi PLHIV, including those with multi-drug resistance. Finally, a full probabilistic sensitivity analysis was not executed, and indeed is not recommended in dynamic transmission modeling due to the implicit objective nature of model calibration to actual practice [23].

The One4All testing algorithm increased the probability of HIV test receipt, and thus accelerated ART uptake among those presenting to hospital in 6 study sites in Guangxi Zhuang Autonomous Region, China. Implementing the strategy in hospitals across the region would be highly cost-effective, but best implemented alongside other public health strategies to seek, test, treat and retain people living with HIV/AIDS.

## Supporting Information

S1 Appendix (DOCX)

## References

[1] Cohen MS, Chen YQ, McCauley M, Gamble T, Hosseinipour MC, et al (2011) Prevention of HIV-1 infection with early antiretroviral therapy. N Engl J Med 365: 493–505. 10.1056/NEJMoa1105243 21767103 PMC3200068

[2] Montaner JS, Lima VD, Barrios R, Yip B, Wood E, et al (2010) Association of highly active antiretroviral therapy coverage, population viral load, and yearly new HIV diagnoses in British Columbia, Canada: a population-based study. Lancet 376: 532–539. 10.1016/S0140-6736(10)60936-1 20638713 PMC2996043

[3] Rodger A, Bruun T, V C, J L, Vernazza P, et al (2014) HIV transmission risk through condomless sex if HIV positive partner is on supressive ART: PARTNER study CROI Boston.

[4] Wood E, Kerr T, Marshall BDL, Li K, Zhang R, et al (2009) Longitudinal community plasma HIV-1-RNA concentrations and incidence of HIV-1 among injecting drug users: a prospective cohort study. BMJ 338: 1191–1194. 10.1136/bmj.b1649 PMC2675696 19406887

[5] Nosyk B, Audoin B, Beyrer C, Cahn P, Granich R, et al (2013) Examining the evidence on the causal effect of HAART on transmission of HIV using the Bradford Hill criteria. AIDS 27: 1159–1165. 10.1097/QAD.0b013e32835f1d68 23902921 PMC4539010

[6] Gardner EM, McLee MP, Steiner JF, del Rio C, Burman WJ (2011) The spectrum of engagement in HIV care and its relevance to test-and-treat strategies for prevention of HIV infection. Clin Infect Dis 52: 793–800. 10.1093/cid/ciq243 21367734 PMC3106261

[7] Nosyk B, Montaner JS, Colley G, Lima VD, Chan K, et al (2014) The cascade of HIV care in British Columbia, Canada, 1996–2011: a population-based retrospective cohort study. The Lancet Infectious diseases 14: 40–49. 10.1016/S1473-3099(13)70254-8 24076277 PMC4017913

[8] UNAIDS (2014) 90-90-90 An ambitious treatment target to help end the AIDS epidemic.

[9] (2011) 2011 Estimates for the HIV/AIDS Epidemic in China. Beijing, China Ministry of Health, People's Republic of China, United Nations and WHO Programme on HIV/AIDS.

[10] (2012) Tabulation On The 2010 Population Census Of Guangxi Zhuang Autonomous Region. Guangxi Zhuang Autonomous Region Bureau of Statistics, Guangxi Zhuang Autonomous Region Population Census Office.

[11] National Center for AIDS/STD Control and Prevention, China CDC (2012) Analysis of HIV/AIDS testing and treatment program in Guangxi Beijing: National Center for AIDS/STD Control and Prevention, China CDC.

[12] Wu Z, Tang Z, Mao Y, VanVeldhuisen P, Ling W, et al. Testing and Linkage to HIV Care in China: A Cluster-Randomized Trial. Submitted to: The Lancet. 10.1016/S2352-3018(17)30131-5 PMC6639122 28867267

[13] (2015) IAPAC Guidelines for Optimizing the HIV Care Continuum for Adults and Adolescents. Journal of the International Association of Providers of AIDS Care 14 Suppl 1: S3–S34. 26527218 10.1177/2325957415613442

[14] Nosyk B, Krebs E, Eyawo O, Min JE, Barrios R, et al (2014) Cost-effectiveness analysis along the continuum of HIV care: how can we optimize the effect of HIV treatment as prevention programs? Curr HIV/AIDS Rep 11: 468–478. 10.1007/s11904-014-0227-7 25173799

[15] Huang YL, Lasry A, Hutchinson AB, Sansom SL (2015) A systematic review on cost effectiveness of HIV prevention interventions in the United States. Appl Health Econ Health Policy 13: 149–156. 10.1007/s40258-014-0142-5 25536927

[16] Bove J, Golden MR, Dhanireddy S, Harrington RD, Dombrowski JC (2015) Outcomes of a Clinic-Based, Surveillance-Informed Intervention to Relink Patients to HIV Care. J Acquir Immune Defic Syndr. 10.1097/QAI.0000000000000707 PMC4607589 26068720

[17] Chaiyachati KH, Ogbuoji O, Price M, Suthar AB, Negussie EK, et al (2014) Interventions to improve adherence to antiretroviral therapy: a rapid systematic review. AIDS 28: S187–204. 10.1097/QAD.0000000000000252 24849479

[18] Gardner LI, Giordano TP, Marks G, Wilson TE, Craw JA, et al (2014) Enhanced personal contact with HIV patients improves retention in primary care: a randomized trial in 6 US HIV clinics. Clin Infect Dis 59: 725–734. 10.1093/cid/ciu357 24837481 PMC4366591

[19] Long EF, Brandeau ML, Owens DK (2010) The cost-effectiveness and population outcomes of expanded HIV screening and antiretroviral treatment in the United States. Ann Intern Med 153: 778–789. 10.7326/0003-4819-153-12-201012210-00004 21173412 PMC3173812

[20] Nosyk B, Min JE, Lima VD, Hogg RS, Montaner JS (2015) Cost-effectiveness of population-level expansion of highly active antiretroviral treatment for HIV in British Columbia, Canada: a modelling study. The lancet HIV 2: e393–400. 10.1016/S2352-3018(15)00127-7 26423553 PMC4610179

[21] Li J, Gilmour S, Zhang H, Koyanagi A, Shibuya K (2012) The epidemiological impact and cost-effectiveness of HIV testing, antiretroviral treatment and harm reduction programs. AIDS 26: 2069–2078. 10.1097/QAD.0b013e3283574e54 22781221

[22] Region SBoGZA (2015) Guangxi Statistical Yearbook-2015. China Statistics Press.

[23] Pitman R, Fisman D, Zaric G S, Postma M, Kretzschmar M, et al (2012) Dynamic transmission modeling: A report of the ISPOR-SMDM modeling good research practices task force-5. Value Health 15: 828–834. 10.1016/j.jval.2012.06.011 22999132 PMC7110742

[24] Mo Y (2010) Researching the Drug Problems in Guangxi's Frontier District: Guangxi University.

[25] Xu Y (2008) Research of psychological and physiological characteristics on male homosexuality: Zhejiang University.

[26] Chow EP, Lau JT, Zhuang X, Zhang X, Wang Y, et al (2014) HIV prevalence trends, risky behaviours, and governmental and community responses to the epidemic among men who have sex with men in China. BioMed research international 2014: 607261 10.1155/2014/607261 24822214 PMC4005141

[27] Chen Y, Chen J, Liu W, Xu G, Wang H, et al (2012) Analysis of high risk behaviors of drug users related to HIV/AIDS transmitting in some area of Guangxi. Chinese Journal of AIDS & STD 18: 86–88.

[28] Wang X, Lan G, Shen Z, Vermund SH, Zhu Q, et al (2014) HIV and syphilis prevalence trends among men who have sex with men in Guangxi, China: yearly cross-sectional surveys, 2008–2012. BMC Infect Dis 14: 367 10.1186/1471-2334-14-367 24993252 PMC4091643

[29] (2016) HIV and AIDS Data Hub for Asia-Pacific. www.aidsdatahub.org.

[30] Chen L (2014) Study on HIV/AIDS Epidemic Characteristics and Mathematical Discriminative Model of Regional Categories in Guangxi: Guangxi Medical University.

[31] Quinn TC, Wawer MJ, Sewankambo N, Serwadda D, Li C, et al (2000) Viral load and heterosexual transmission of human immunodeficiency virus type 1. Rakai Project Study Group. N Engl J Med 342: 921–929. 10.1056/NEJM200003303421303 10738050

[32] Abbas UL, Anderson RM, Mellors JW (2006) Potential impact of antiretroviral therapy on HIV-1 transmission and AIDS mortality in resource-limited settings. J Acquir Immune Defic Syndr 41: 632–641. 10.1097/01.qai.0000194234.31078.bf 16652038

[33] Downs AM, De Vincenzi I (1996) Probability of heterosexual transmission of HIV: relationship to the number of unprotected sexual contacts. European Study Group in Heterosexual Transmission of HIV. J Acquir Immune Defic Syndr Hum Retrovirol 11: 388–395. 8601226 10.1097/00042560-199604010-00010

[34] Hollingsworth TD, Anderson RM, Fraser C (2008) HIV-1 transmission, by stage of infection. J Infect Dis 198: 687–693. 10.1086/590501 18662132

[35] Padian NS, Shiboski SC, Glass SO, Vittinghoff E (1997) Heterosexual transmission of human immunodeficiency virus (HIV) in northern California: results from a ten-year study. Am J Epidemiol 146: 350–357. 9270414 10.1093/oxfordjournals.aje.a009276

[36] Wawer MJ, Gray RH, Sewankambo NK, Serwadda D, Li X, et al (2005) Rates of HIV-1 transmission per coital act, by stage of HIV-1 infection, in Rakai, Uganda. J Infect Dis 191: 1403–1409. 10.1086/429411 15809897

[37] Mastro TD, de Vincenzi I (1996) Probabilities of sexual HIV-1 transmission. AIDS 10 Suppl A: S75–82. 10.1097/00002030-199601001-00011 8883613

[38] Caceres CF, van Griensven GJ (1994) Male homosexual transmission of HIV-1. AIDS 8: 1051–1061. 7986400 10.1097/00002030-199408000-00004

[39] Jacquez JA, Koopman JS, Simon CP, Longini IM Jr. (1994) Role of the primary infection in epidemics of HIV infection in gay cohorts. J Acquir Immune Defic Syndr 7: 1169–1184. 7932084

[40] Vittinghoff E, Douglas J, Judson F, McKirnan D, MacQueen K, et al (1999) Per-contact risk of human immunodeficiency virus transmission between male sexual partners. Am J Epidemiol 150: 306–311. 10430236 10.1093/oxfordjournals.aje.a010003

[41] Wang S, Moss JR, Hiller JE (2011) The cost-effectiveness of HIV voluntary counseling and testing in China. Asia Pac J Public Health 23: 620–633. 10.1177/1010539511412576 21727082

[42] Wei L, Chen J, Rodolph M, Beauchamp G, Masse B, et al (2006) HIV incidence, retention, and changes of high-risk behaviors among rural injection drug users in Guangxi, China. Subst Abus 27: 53–61. 10.1300/j465v27n04_07 17347126

[43] Wu Z, Xu J, Liu E, Mao Y, Xiao Y, et al (2013) HIV and syphilis prevalence among men who have sex with men: a cross-sectional survey of 61 cities in China. Clin Infect Dis 57: 298–309. 10.1093/cid/cit210 23580732 PMC3689345

[44] Chen Y, Tang Z, Shen Z, Zhu Q, Liang F, et al (2013) Trend of HIV/AIDS Epidemic among Drug User in Guangxi Zhuang Autonomous Region, 2007–2012. Disease Surveillance 28: 643–647.

[45] She Y, Zhong X, Zhang Y, Hao B, Liang H, et al (2010) Knowledge and Behaviors Related to HIV/AIDS among Men who Have Sex with Men in Western China. Journal of Chongqing Medical University 135: 1902–1905.

[46] Davis KR, Weller SC (1999) The effectiveness of condoms in reducing heterosexual transmission of HIV. Fam Plann Perspect 31: 272–279. 10614517

[47] Giannou FK, Tsiara CG, Nikolopoulos GK, Talias M, Benetou V, et al (2015) Condom effectiveness in reducing heterosexual HIV transmission: a systematic review and meta-analysis of studies on HIV serodiscordant couples. Expert Rev Pharmacoecon Outcomes Res: 1–11. 10.1586/14737167.2016.1102635 26488070

[48] Cayley WE Jr. (2004) Effectiveness of condoms in reducing heterosexual transmission of HIV. Am Fam Physician 70: 1268–1269. 15508535

[49] Jack B. Homer CLSC (1991) A Model of HIV Transmission through Needle Sharing. Interfaces 21: 26–49.

[50] Zaric GS, Barnett PG, Brandeau ML (2000) HIV transmission and the cost-effectiveness of methadone maintenance. Am J Public Health 90: 1100–1111. 10897189 10.2105/ajph.90.7.1100 PMC1446290

[51] Wang X, Ge X, Tang Z, Shen Z, Lan W, et al (2015) Epidemiological Characteristics of HIV/AIDS in Guangxi during 2008–2013. J Applied Prev Med 21: 171–222.

[52] Zhang J, Yu KF (1998) What's the relative risk? A method of correcting the odds ratio in cohort studies of common outcomes. JAMA 280: 1690–1691. 9832001 10.1001/jama.280.19.1690

[53] Sanders GD, Bayoumi AM, Sundaram V, Bilir SP, Neukermans CP, et al (2005) Cost-effectiveness of screening for HIV in the era of highly active antiretroviral therapy. N Engl J Med 352: 570–585. 10.1056/NEJMsa042657 15703422

[54] Kamb ML, Fishbein M, Douglas JM Jr, Rhodes F, Rogers J, et al (1998) Efficacy of risk-reduction counseling to prevent human immunodeficiency virus and sexually transmitted diseases: a randomized controlled trial. JAMA 280: 1161–1167. 9777816 10.1001/jama.280.13.1161

[55] (2010) China 2010 UNGASS Country Progress Report (2008–2009). Ministry of Health of the People’s Republic of China.

[56] Mellors JW, Muñoz A, Giorgi JV, Margolick JB, Tassoni CJ, et al (1997) Plasma viral load and CD4+ lymphocytes as prognostic markers of HIV-1 infection. Ann Intern Med 126: 946–954. 9182471 10.7326/0003-4819-126-12-199706150-00003

[57] Vlahov D, Graham N, Hoover D, Flynn C, Bartlett JG, et al (1998) Prognostic indicators for AIDS and infectious disease death in HIV-infected injection drug users: plasma viral load and CD4+ cell count. JAMA 279: 35–40. 9424041 10.1001/jama.279.1.35

[58] Hughes MD, Johnson VA, Hirsch MS, Bremer JW, Elbeik T, et al (1997) Monitoring plasma HIV-1 RNA levels in addition to CD4+ lymphocyte count improves assessment of antiretroviral therapeutic response. ACTG 241 Protocol Virology Substudy Team. Ann Intern Med 126: 929–938. 9182469 10.7326/0003-4819-126-12-199706150-00001

[59] Holtgrave DR, Pinkerton SD (1997) Updates of cost of illness and quality of life estimates for use in economic evaluations of HIV prevention programs. J Acquir Immune Defic Syndr Hum Retrovirol 16: 54–62. 9377126 10.1097/00042560-199709010-00009

[60] Honiden S, Sundaram V, Nease RF, Holodniy M, Lazzeroni LC, et al (2006) The effect of diagnosis with HIV infection on health-related quality of Life. Qual Life Res 15: 69–82. 10.1007/s11136-005-8485-x 16411032

[61] Schackman BR, Goldie SJ, Freedberg KA, Losina E, Brazier J, et al (2002) Comparison of health state utilities using community and patient preference weights derived from a survey of patients with HIV/AIDS. Med Decis Making 22: 27–38. 11833663 10.1177/0272989X0202200103

[62] Tengs TO, Lin TH (2002) A meta-analysis of utility estimates for HIV/AIDS. Med Decis Making 22: 475–481. 12458977 10.1177/0272989X02238300

[63] Zaric GS, Barnett PG, Brandeau ML (2000) HIV transmission and the cost-effectiveness of methadone maintenance. Am J Public Health 90: 1100–1111. 10897189 10.2105/ajph.90.7.1100 PMC1446290

[64] Long EF, Brandeau ML, Galvin CM, Vinichenko T, Tole SP, et al (2006) Effectiveness and cost-effectiveness of strategies to expand antiretroviral therapy in St. Petersburg, Russia. AIDS 20: 2207–2215. 10.1097/QAD.0b013e328010c7d0 17086061

[65] Yang HM, Li J, Wu ZY, Xu LZ, Wang KA (2003) [Study on the utilization of health services and costs of hospital-based medical care for 29 patients with HIV/AIDS in China]. Zhonghua liu xing bing xue za zhi = Zhonghua liuxingbingxue zazhi 24: 393–396. 12820935

[66] Nosyk B, Lima V, Colley G, Yip B, Hogg RS, et al (2014) Costs of health resource utilization among HIV-positive individuals in British Columbia, Canada: Results from a population-level study. PharmacoEconomics. 10.1007/s40273-014-0229-8 PMC4677778 25404425

[67] Cheng G, Qian ZH, Hu J (2009) [Longitudinal analysis of technical efficiency of voluntary counseling and testing of HIV in China]. Beijing da xue xue bao Yi xue ban = Journal of Peking University Health sciences 41: 135–140. 19377617

[68] Hyle EP, Jani IV, Lehe J, Su AE, Wood R, et al (2014) The clinical and economic impact of point-of-care CD4 testing in mozambique and other resource-limited settings: a cost-effectiveness analysis. PLoS medicine 11: e1001725 10.1371/journal.pmed.1001725 25225800 PMC4165752

[69] Ciaranello AL, Myer L, Kelly K, Christensen S, Daskilewicz K, et al (2015) Point-of-care CD4 testing to inform selection of antiretroviral medications in south african antenatal clinics: a cost-effectiveness analysis. PLoS One 10: e0117751 10.1371/journal.pone.0117751 25756498 PMC4355621

[70] Bolker BM, Brooks ME, Clark CJ, Geange SW, Poulsen JR, et al (2009) Generalized linear mixed models: a practical guide for ecology and evolution. Trends in ecology & evolution 24: 127–135. 19185386 10.1016/j.tree.2008.10.008

[71] NCAIDS N, China CDC (2011–2014) Update on the AIDS/STD epidemic in China and main response in control and prevention. Chinese Journal of AIDS&STD.

[72] Nosyk B, Min J, Lima VD, Yip B, Hogg RS, et al (2013) HIV-1 disease progression during highly active antiretroviral therapy: an application using population-level data in British Columbia: 1996–2011. Journal of acquired immune deficiency syndromes 63: 653–659. 10.1097/QAI.0b013e3182976891 24135777 PMC3800281

[73] Fryback DG, Dasbach EJ, Klein R, Klein BE, Dorn N, et al (1993) The Beaver Dam Health Outcomes Study: initial catalog of health-state quality factors. Med Decis Making 13: 89–102. 8483408 10.1177/0272989X9301300202

[74] GDP per capita (current US$). The World Bank.

[75] World Health Organization (2015) Choosing interventions that are cost-effective (WHOCHOICE): cost-effectiveness thresholds.

[76] Schackman BR, Metsch LR, Colfax GN, Leff JA, Wong A, et al (2013) The cost-effectiveness of rapid HIV testing in substance abuse treatment: Results of a randomized trial. Drug and Alcohol Dependence 128: 90–97. 10.1016/j.drugalcdep.2012.08.009 22971593 PMC3546145

[77] Nosyk B, Krebs E, Min JE, Ahamed K, Buxton J, et al (2015) The 'Expanded HIV care in opioid substitution treatment' (EHOST) cluster-randomized, stepped-wedge trial: A study protocol. Contemporary clinical trials 45(Pt B): 9. 10.1016/j.cct.2015.08.020 26342295

[78] Paltiel AD, Weinstein MC, Kimmel AD, Seage GR III, Losina E, et al (2005) Expanded screening for HIV in the United States—an analysis of cost-effectiveness. N Engl J Med 352: 586–595. 10.1056/NEJMsa042088 15703423

[79] Walensky RP, Freedberg KA, Weinstein MC, Paltiel AD (2007) Cost-effectiveness of HIV testing and treatment in the United States. Clinical infectious diseases: an official publication of the Infectious Diseases Society of America 45 Suppl 4: S248–254. 18190295 10.1086/522546 PMC2365915

[80] Des Jarlais DC, Arasteh K, McKnight C, Hagan H, Perlman DC, et al (2010) HIV infection during limited versus combined HIV prevention programs for IDUs in New York City: the importance of transmission behaviors. Drug Alcohol Depend 109: 154–160. 10.1016/j.drugalcdep.2009.12.028 20163922 PMC4447191

[81] Jones A, Cremin I, Abdullah F, Idoko J, Cherutich P, et al (2014) Transformation of HIV from pandemic to low-endemic levels: a public health approach to combination prevention. Lancet 384: 272–279. 10.1016/S0140-6736(13)62230-8 24740087

[82] Strathdee SA, Shoptaw S, Dyer TP, Quan VM, Aramrattana A, et al (2012) Towards combination HIV prevention for injection drug users: addressing addictophobia, apathy and inattention. Curr Opin HIV AIDS 7: 320–325. 10.1097/COH.0b013e32835369ad 22498479 PMC3646543

[83] Vermund SH, Hayes RJ (2013) Combination prevention: new hope for stopping the epidemic. Curr HIV/AIDS Rep 10: 169–186. 10.1007/s11904-013-0155-y 23456730 PMC3642362

[84] Chang LW, Serwadda D, Quinn TC, Wawer MJ, Gray RH, et al (2013) Combination implementation for HIV prevention: moving from clinical trial evidence to population-level effects. Lancet Infect Dis 13: 65–76. 10.1016/S1473-3099(12)70273-6 23257232 PMC3792852

[85] Anderson SJ, Cherutich P, Kilonzo N, Cremin I, Fecht D, et al (2014) Maximising the effect of combination HIV prevention through prioritisation of the people and places in greatest need: a modelling study. Lancet 384: 249–256. 10.1016/S0140-6736(14)61053-9 25042235

[86] The HIV Modelling Consortium Treatment as Prevention Editorial Writing Group (2012) HIV Treatment as Prevention: models, data, and questions–towards evidence-based decision-making. PLoS Medicine 9: e1001259 10.1371/journal.pmed.1001259 22802739 PMC3393655

[87] Eaton JW, Hallett TB (2014) Why the proportion of transmission during early-stage HIV infection does not predict the long-term impact of treatment on HIV incidence. PNAS 111: 16202–16207. 10.1073/pnas.1323007111 25313068 PMC4234601

