# Supplementary material for: Cost-Effectiveness of the ‘One4All’ HIV Linkage Intervention in Guangxi Zhuang Autonomous Region, China
Source: PLoS One. 2016 Nov 28;11(11):e0167308. doi: 10.1371/journal.pone.0167308 (PMC5125690; doi:10.1371/journal.pone.0167308)
Supplement: S1 Appendix — (DOCX) [file pone.0167308.s001.docx]

**Supplementary Appendix for “Cost-effectiveness of the ‘One4All’ HIV linkage intervention in Guangxi Zhuang Autonomous Region, China”**

1. **Model Description**

*1.1 Differential Equations*

We created the following system of nonlinear differential equations of each of the four risk groups considered in our model. The complete model is thus comprised of 60 equations (4 risk groups x 15). We present the equations for risk group k. We denote S_i_, I_i_, D_i_, and T_i_ to represent model classes capturing individuals susceptible to HIV infection (S_1_, unscreened and S_2_, screened for HIV), HIV infected, diagnosed, and on antiretroviral treatment (ART), respectively, in each CD4-based stage of HIV progression i = {CD4≥500 cells/mm^3^, 350≤CD4<500, 200≤CD4<350, CD4<200} for I_i_, D_i_, and T_i_. We let $X_{j}$denote $X_{jt}$, representing number of people in compartment *j* at time *t*.

1. $\frac{dX_{S_{1}}}{dt}=\rho\sum_{\forall i} X_{i}+ \omega X_{S_{2}}- \psi_{k}X_{S_{1}}-({\sum_{j\neq S_{1}, S_{2}} \lambda_{S_{1},j\left( t \right)})X_{S_{1}} -\mu}_{S_{k}}X_{S_{1}}$
2. $\frac{dX_{S_{2}}}{dt}=\psi_{k}X_{S_{1}}- \omega X_{S_{2}} -({\sum_{j\neq S_{1}, S_{2}} \lambda_{S_{2},j(t)})X_{S_{2}} -\mu}_{S_{k}}X_{S_{2}}$
3. $\frac{dX_{I_{1}}}{dt}=\left( \sum_{j\neq S_{1}, S_{2}} \lambda_{S_{1},j\left( t \right)} \right)X_{S_{1}}+\left( \sum_{j\neq S_{1}, S_{2}} \lambda_{S_{2},j\left( t \right)} \right)X_{S_{2}}-{\psi_{k}X}_{I_{1}}-\theta_{\overline{T}_{1}}X_{I_{1}}- \mu_{\overline{T}_{1}}X_{I_{1}}$
4. $\frac{dX_{I_{2}}}{dt}=\theta_{\overline{T}_{1}}X_{I_{1}}-{\psi_{k}X}_{I_{2}}-\theta_{\overline{T}_{2}}X_{I_{2}}- \mu_{\overline{T}_{2}}X_{I_{2}}$
5. $\frac{dX_{I_{3}}}{dt}=\theta_{\overline{T}_{2}}X_{I_{2}}-(\psi_{k}+\nu_{3})X_{I_{3}}-\theta_{\overline{T}_{3}}X_{I_{3}}- \mu_{\overline{T}_{3}}X_{I_{3}}$
6. $\frac{dX_{I_{4}}}{dt}=\theta_{\overline{T}_{3}}X_{I_{3}}-(\psi_{k}+\nu_{4})X_{I_{4}}- \mu_{\overline{T}_{4}}X_{I_{4}}$
7. $\frac{dX_{D_{1}}}{dt}=\theta_{T_{1,}D_{1}(t)}X_{T_{1}}+{\psi_{k}X}_{I_{1}}-\theta_{\overline{T}_{1}}X_{D_{1}}- \mu_{\overline{T}_{1}}X_{D_{1}}$
8. $\frac{dX_{D_{2}}}{dt}=\theta_{T_{2,}D_{2}(t)}X_{T_{2}}+\theta_{\overline{T}_{1}}X_{D_{1}}+ {\psi_{k}X}_{I_{2}} -\theta_{\overline{T}_{2}}X_{D_{2}}-\mu_{\overline{T}_{2}}X_{D_{2}}$
9. $\frac{dX_{D_{3}}}{dt}={\theta_{T_{3,}D_{3}(t)}X_{T_{3}}+\theta}_{\overline{T}_{2}}X_{D_{2}}+ {(\psi_{k}+\nu_{3})(1-\varphi)X}_{I_{3}} -\theta_{\overline{T}_{3}}X_{D_{3}}- \alpha_{t}X_{D_{3}}-\mu_{\overline{T}_{3}}X_{D_{3}}$
10. $\frac{dX_{D_{4}}}{dt}={\theta_{T_{4,}D_{4}(t)}X_{T_{4}}+\theta}_{\overline{T}_{3}}X_{D_{3}}+ (\psi_{k}+\nu_{4})(1-\varphi)X_{I_{4}} - \alpha_{t}X_{D_{4}}-\mu_{\overline{T}_{4}}X_{D_{4}}$
11. $\frac{dX_{T_{1}}}{dt}=\sum_{j\neq1} \theta_{T_{j,}T_{1}(t)}X_{T_{j}}- \sum_{i\neq1} \theta_{T_{1,}T_{i}(t)}X_{T_{1}}-\theta_{T_{1,}D_{1}(t)}X_{T_{1}}-\mu_{T_{1}}X_{T_{1}}$
12. $\frac{dX_{T_{2}}}{dt}=\sum_{j\neq2} \theta_{T_{j,}T_{2}(t)}X_{T_{j}}- \sum_{i\neq2} \theta_{T_{2,}T_{i}\left( t \right)}X_{T_{2}}-\theta_{T_{2,}D_{2}(t)}X_{T_{2}}-\mu_{T_{2}}X_{T_{2}}$
13. $\frac{dX_{T_{3}}}{dt}=\sum_{j\neq3} \theta_{T_{j,}T_{3}(t)}X_{T_{j}}+\alpha_{t}X_{D_{3}}+(\psi_{k}+\nu_{3})\varphi X_{I_{3}}- \sum_{i\neq3} \theta_{T_{3,}T_{i}\left( t \right)}X_{T_{3}}-\theta_{T_{3,}D_{3}(t)}X_{T_{3}}-\mu_{T_{3}}X_{T_{3}}$
14. $\frac{dX_{T_{4}}}{dt}=\sum_{j\neq4} \theta_{T_{j,}T_{4}(t)}X_{T_{j}}+\alpha_{t}X_{D_{4}}+(\psi_{k}+\nu_{4})\varphi X_{I_{4}}- \sum_{i\neq4} \theta_{T_{4,}T_{i}\left( t \right)}X_{T_{4}}-\theta_{T_{4,}D_{4}(t)}X_{T_{4}}-\mu_{T_{4}}X_{T_{4}}$
15. $\frac{dX_{D}}{dt}=\mu_{S_{k}}(X_{S_{1}}+X_{S_{2}})+\sum_{i} \mu_{\overline{T}_{i}}(X_{I_{i}}+ X_{D_{i}}) + \sum_{i} \mu_{T_{it}}X_{T_{i}}$

A schematic representation of the model is shown in Figure A1. In the diagram, boxes represent cohorts of individuals, stratified by HIV status, identification (ie. screening) status, and diagnosis and treatment status if infected. Arrows represent possible transitions between compartments. Individuals move from susceptible (screened or not screened) to infected health states through the total contact rate, or force of infection ($\lambda_{ij})$, for susceptible individuals in state i, from infected individuals in state j; from infected to diagnosed through screening ($\psi_{k}$) and symptom-based case finding (*ν_i_*), and a portion $\varphi$ directly towards treatment (only for eligible infectious individuals with CD4 <350 cells/mm^3^); from diagnosed to treated according to the calibrated probability of treatment initiation ($\alpha_{t}$) when meeting eligibility criteria (CD4 <350 cells/mm^3^) [[1](#_ENREF_1)]. Once in treatment, individuals could transition to any of the other treatment states $j\neq i$ ($\sum_{j\neq i} \theta_{T_{j,it}T_{i}(t)}X_{T_{j}}T_{j})$, or drop out of treatment while staying at the same CD4 level ($\theta_{T_{i,Dt}D_{i}(t)}{X_{T_{i}}T}_{i})$. Although not shown, individuals may also leave each compartment according to the mortality and/or maturation rate. Descriptions and valuations of the key parameters dictating model dynamics are presented in Table A1.

We note that the One4All screening intervention will lead to the increase in the monthly screening rate and solely for heterosexual individuals with CD4 <350 cells/mm^3^, corresponding to the primary results of CTN-0056 trial-“Testing and Linkage to HIV Care in China”. The CTN-0056 trial demonstrated the effectiveness of proposed One4All screening intervention conducted in 12 county hospitals in Guangxi Zhuang Autonomous Region, China, that participants were predominantly heterosexual (>97%) individuals in Guangxi with progressed disease state (median CD4 counts<200 cells/mm^3^) who actively sought for HIV testing and care and were screened positive on an initial HIV enzyme immunoassay.

The number of individuals entering the pool of susceptible individuals was proportional to the average Guangxi population growth during the study period, and represented by $\rho_{i}$. Death was an 'absorbing' health state, with clients from each state transitioning in each model cycle (equation 15). The full set of parameters dictating movement between compartments is detailed in Table A1.

The ‘trace’ of the model, capturing the number of individuals infected, diagnosed and in treatment in each calendar month over time and across scenarios (standard of care and One4All screening intervention) is presented in Figure A2. With the One4All intervention implemented across the region, our analysis estimated higher proportions of individuals accessing treatment, and lower proportions of undiagnosed people living with HIV (PLHIV) compared to the current standard of care.

*1.2 HIV Transmission*

The dynamic model captures HIV transmission via three modes: heterosexual contact, homosexual contact, and needle-sharing. Table A2 shows the possible modes of transmission between any two risk groups. Consistent with prior studies, we allowed MSM to potentially have heterosexual contact with women.

For each risk group we assumed one average value for the number of same-sex and opposite-sex partners, adjusting for HIV-infection status, which reduces their number of sexual partners due to screening and counseling. Annualized figures were adjusted to represent monthly figures using the following formula: (1 - (1 - p)^1/12^).

The sufficient contact rate between uninfected and infected individuals is represented as a matrix, λ = $\lambda_{ij}$, representing the sufficient contact rate between members of (uninfected) compartment i and members of (infected) compartment j. We calculated the total contact rate,$\lambda_{ij}$, as the sum of the three transmission model: needle-sharing ($\gamma_{ij}$), heterosexual contact ($\beta_{ij}^{o}$), and homosexual contact ($\beta_{ij}^{s}$). We calculated the overall sufficient contact rate between uninfected individuals in compartment i and infected individuals in compartment j by first converting the annual transmission probability to a continuous rate, according to the formula: $rate=\left\{ -ln\left( 1-p \right) \right\}/t$. We then summed over the three modes of transmission: needle-sharing ($\gamma_{ij}$), heterosexual contact ($\beta_{ij}^{o}$), and homosexual contact ($\beta_{ij}^{s}$). For small probability values, we used the approximation $p\approx-ln(1-p)$. The total contact rate at time t between individuals in compartments i and j, $\lambda_{ijt}$, is:

$$\lambda_{ijt}=\gamma_{ijt}+\beta_{ijt}^{o}+\beta_{ijt}^{s}$$

The full set of parameters used to estimate HIV transmission within and between risk groups was presented in Table A3. We note that in order to adequately characterize changes in HIV risk behaviours (shared drug injection and condom use) observed during the model calibration period (2011-2014), we used data on (i) the annual number of individuals accessing methadone maintenance treatment (MMT) [[2](#_ENREF_2)] (ii) the total non-HIV sexually transmitted disease (STD) rate [[2](#_ENREF_2)] within China, both throughout the study period.

The number of injections per month (200/12=16.67) [[3](#_ENREF_3)] was multiplied by the (fixed) probability of shared injection (0.264) [[4](#_ENREF_4)] and the inverse of the MMT initiation index (Figure A3, Panel A), to derive the time-dependent number of shared injections (Figure A3, Panel B). Similarly, the rate (per 100,000 individuals) of non-HIV STDs was calculated as the sum of rates of new gonorrhea and syphilis infections, as reported through monthly report of updates in AIDS/STD by NCAIDS, NCSTD and China CDC [[2](#_ENREF_2)] (Figure A3, Panel C). The probability of condom use [[5](#_ENREF_5),[6](#_ENREF_6),[7](#_ENREF_7)] was multiplied by the inverse of the STD rate to solve for the probability of condom use amongst sexual partnerships between individuals in MSM and Heterosexual compartments over time (Figure A3, Panel D).

1.2.1 Needle-sharing transmission: The needle-sharing sufficient contact rate between uninfected individuals in compartment i and infected individuals in compartment j at time t is:

$$\gamma_{ijt}=1-\left( 1-\left[ \frac{X_{jt}d_{j}s_{j}}{\sum_{k} X_{kt}d_{k}s_{k}} \right]\tau_{ij} \right)^{d_{i}s_{i}}$$

Where i, j, k correspond to compartments of IDUs. The term in brackets, $\left[ \frac{X_{jt}d_{j}s_{j}}{\sum_{k} X_{kt}d_{k}s_{k}} \right]$, corresponds to the probability of selecting a needle-sharing partner in compartment j, based on a proportional mixing assumption (ie. individuals with many partners are more likely to select a partner who also has many partners). We note that the probability of transmission is reduced by 50% ($\delta_{I}$) when the partner in compartment j is on ART [[3](#_ENREF_3),[8](#_ENREF_8)].

1.2.2 Heterosexual transmission: The heterosexual sufficient contact rate between uninfected individuals in compartment i and infected individuals in compartment j is:

$$\beta_{ijt}^{o}=1-\left( 1-\left[ \frac{X_{jt}n_{j}^{o}(1-u_{j}^{o}\kappa)}{\sum_{k} X_{kt}n_{k}^{o}(1-u_{k}^{o}\kappa)} \right]\sigma_{ij} \right)^{n_{i}^{o}(1-u_{i}^{o}\kappa)}$$

Where i is male and j,k are female, or vice versa. We note that for the sake of parsimony, we did not create separate compartments for female IDU and heterosexuals; instead, we adjusted the number of sexual partnerships between heterosexuals, injection drug users and MSM, heterosexuals and MSM, and heterosexuals and MSM/IDU by a factor of 0.5. The term in brackets, $\left[ \frac{X_{jt}n_{j}^{o}(1-u_{j}^{o}\kappa)}{\sum_{k} X_{kt}n_{k}^{o}(1-u_{k}^{o}\kappa)} \right]$, corresponds to the probability of selecting a sexual partner in compartment j. We note that the number of sexual partners of individuals in compartment j is reduced by 20% and 90% (ε) for HIV and AIDS patients respectively following diagnosis [[3](#_ENREF_3),[9](#_ENREF_9),[10](#_ENREF_10),[11](#_ENREF_11),[12](#_ENREF_12)], and the probability of transmission is reduced by 90% ($\delta_{M}$) when the partner in compartment j is on ART [[13](#_ENREF_13),[14](#_ENREF_14),[15](#_ENREF_15)].

1.2.3 Homosexual transmission: The homosexual sufficient contact rate between uninfected individuals in compartment i and infected individuals in compartment j is:

$$\beta_{ijt}^{s}=1-\left( 1-\left[ \frac{X_{jt}n_{j}^{s}(1-u_{j}^{s}\kappa)}{\sum_{k} X_{kt}n_{k}^{s}(1-u_{k}^{s}\kappa)} \right]\sigma_{ij} \right)^{n_{i}^{s}(1-u_{i}^{s}\kappa)}$$

Where i, j, k correspond to compartments of MSM. The term in brackets, $\left[ \frac{X_{jt}n_{j}^{s}(1-u_{j}^{s}\kappa)}{\sum_{k} X_{kt}n_{k}^{s}(1-u_{k}^{s})} \right]$, again corresponds to the probability of selecting a sexual partner in compartment j. We note that the number of sexual partners of individuals in compartment j is reduced by 20% and 90% (ε) for HIV and AIDS patients respectively following diagnosis [[3](#_ENREF_3),[9](#_ENREF_9),[10](#_ENREF_10),[11](#_ENREF_11),[12](#_ENREF_12)], and the probability of transmission is reduced by 90% ($\delta_{H}$) [[13](#_ENREF_13),[14](#_ENREF_14),[15](#_ENREF_15)] when the partner in compartment j is on ART.

*1.3 Entry, Maturation and Mortality*

We calculated entry, maturation and mortality rates for each of the study risk groups according to the numbers of individuals who enter or mature out of the population each month, as well as published mortality estimates for non-IDU and IDU. The entry probability was derived from the proportion of 15 year olds entering the population, and converted to a rate, incorporating growth in this proportion during the study period. Similarly, the maturation probability was the proportion of 65 year olds within the study population, and converted to a rate. Both baseline entry and maturation rate are derived from provincial Statistical Yearbook [[16](#_ENREF_16)].

As disparate mortality rates substantially changed the proportions of susceptible individuals over time, we adjusted the entry rate of individuals across risk groups (MSM, IDU, MSM/IDU and Hetero) to ensure relatively constant proportions throughout the study period. Entry and maturation was confined to ‘susceptible’ (HIV-negative) states, while mortality rates were applied also to HIV positive states [[17](#_ENREF_17),[18](#_ENREF_18),[19](#_ENREF_19),[20](#_ENREF_20)]. For HIV positive individuals, a broader age group, 15-94 years, was taken into account, corresponded to the findings of *Wang et al.*[[21](#_ENREF_21)] that noticeable increase of new diagnosed cases (approximately 15% from 2008-2013) were identified among individuals aged between 65 to 95 years. As a result, background (non-HIV related) mortality was modified according to the proportion of age group (15-64 years and 65-95 years) and their corresponding mortality rates. Besides the background mortality rate, additional HIV-related mortality rate was also applied, varied for different CD4 strata, while HIV-related mortality rate remained the same for infected and diagnosed PLHIV not on ART and lower for those receiving treatment. Complete data on all-cause mortality was incorporated into the model (Section 2.1).

*1.4 HIV Screening and Symptom-based Case-finding Rates*

Furthermore, observed HIV screening rates in Guangxi Zhuang Autonomous Region, derived from *Wang et al*. [[21](#_ENREF_21)], were incorporated into the model (Figure A4). Specifically, we divided the total annual number of unique test episodes (both nominal and non-nominal tests) by the total population, to get an annual testing rate for the general population. As the data we received were aggregated for the entire region, we assumed high-risk individuals (IDU, MSM and MSM/IDU) were more likely to be screened than those not in high risk groups, consistent with prior literature [[22](#_ENREF_22)]. Thus, calibration was utilized to adjust excessive screening rate for high-risk population. Using this information we derived annual high- and low-risk testing rates, ensuring their sum was equal to the observed provincial estimates. Furthermore, in accordance with the primary results of CTN-0056 project and by converting odds ratio into relative risk [[23](#_ENREF_23)], the monthly screening rate for the heterosexual group was 3.45 times higher than standard of care for the proposed One4All prompt screening intervention scenario. We assume this intervention, if fully implemented, will lead to decrease in the proportion of undiagnosed infections as well as an increase in treatment access, resulting in more controllable and less transmissible HIV epidemic in Guangxi in the long run. Symptom-based case finding rates for low- and high-risk individuals by CD4 stratum (<200, 200-349) were derived from the published literature and were assumed constant throughout the study period.

*1.5 Model Calibration*

We simulated the HIV epidemic by first instantiating the risk group size and HIV prevalence levels based on end of 2010 Guangxi data and population figures from literature, the public health agency of China and Guangxi statistics [[4](#_ENREF_4),[16](#_ENREF_16),[24](#_ENREF_24),[25](#_ENREF_25),[26](#_ENREF_26),[27](#_ENREF_27),[28](#_ENREF_28),[29](#_ENREF_29),[30](#_ENREF_30)], thus using 2010 as an instantiation period for the model, with 2011-2014 as the calibration period. Starting values by risk group, HIV infection level and stage of disease progression are provided in Table A4. We calibrated our model to replicate the true observed annual number of new diagnoses as well as the annual number of PLHIV on ART in Guangxi from 2011-2014 by adjusting the ratio of monthly screening rate for high (IDU, MSM and MSM/IDU) versus low (Hetero) risk group in compartment i ($\psi_{i}^{h}$/$\psi_{i}^{l}$) and probability that a diagnosed individual in diagnosed compartment i entered treatment at a given CD4-based state of disease progression at time t ($\alpha_{it}$) respectively. Results of the calibration were presented in Figure A5. The model-estimated annual number of newly diagnosed cases was within 1% of observed new diagnoses (Panel A). The model-estimated number of PLHIV on ART was within 1% of observed PLHIV on ART at the endpoint of each calendar year (Panel B).

We note that, given the complexities of accurate parameter estimation and inherent gaps in our knowledge and data systems, model calibration is of substantial importance. Well-calibrated models may force the re-estimation of uncertain or implausible parameters, and calibration may be used to generate plausible parameter values when no empirical estimates are available. Furthermore, well-calibrated models that reproduce observed disease incidence, trends, or natural history are extremely important for the establishment of a model’s credibility with decision makers [[31](#_ENREF_31),[32](#_ENREF_32)]. We calibrated our model iteratively, adjusting key parameters that were measured with uncertainty, or for which time-varying data was not available, according to the results of the model validation.

*1.6 Model Validation*

Following calibration to match the known annual number of new diagnosis and number PLHIV on ART at the end of each calendar year, the model was validated to ensure key epidemiological parameters approximated known or externally-estimated figures. We focused on annual mortality among diagnosed PLHIV and the size of the HIV-negative population (aged 15-64). The age constraints imposed are common practice in HIV modeling efforts and implicitly account for the substantially higher levels of HIV risk behaviours pertained to these age groups.

Results of the model validation were presented in Figure A6. Estimated annual mortality among diagnosed PLHIV (at annul endpoint) was on average within 0.66% of public health records of Guangxi CDC during the calibration period (2011-2014) and all within 3% except year 2013 (Panel A). Similarly, the estimated size of HIV-negative population aged 15-64 years old was within 1% of the Guangxi provincial annual statistical yearbook estimated size of this age group, including the baseline year 2010 (Panel B).

**2.0 Model Parameterization**

The model was developed with a combination of a primary analysis on the HIV surveillance database from Guangxi CDC as well as published local and national data. Details on methods used to estimate disease progression and cost parameters are provided below.

*2.1 Disease Progression*

A parametric, continuous-time, Multi-state Markov model (MSMM) was implemented to estimate the impact of prognostic factors on CD4 disease progression and to estimate CD4 state transition probabilities over time. Markov chains constitute a common way of modeling the progression of a chronic disease through various severity states. For these models, a transition matrix with the probabilities of moving from one state to another for a specific time interval is usually estimated from observational cohort data. The time between CD4 measurements is inherently controlled for in this methodology. These models efficiently handle heavily censored data, such as when the exact time of disease onset is unknown or when a subject is observed over a portion of his/her disease history [[33](#_ENREF_33),[34](#_ENREF_34),[35](#_ENREF_35),[36](#_ENREF_36),[37](#_ENREF_37),[38](#_ENREF_38)]. The reasoning and procedure is detailed in a prior publication conducted in British Columbia (BC), Canada [[39](#_ENREF_39)]. We analyzed longitudinal records of CD4 measurements, acquired from Guangxi CDC, NCAIDS and China CDC (Table A5, A6), on 42,267 PLHIV receiving ART to generate a disease progression probability matrix for PLHIV when receiving ART (Table A7). All these 42,267 individuals started their first antiretroviral treatment from 2003 to 2014 with median follow-up of 2.68 (IQR: 1.38-4.43) years, producing a total of 302,208 observations.

The baseline characteristics, including baseline CD4 measurements, of the 42,267 on-ART individuals were summarized in Table A5. Table A6 displays the recorded 302,208 transitions that were used to populate proposed MSMM to calculate monthly transition probabilities between CD4-based disease progression, as well as treatment discontinuation and mortality. We applied MSMM to estimate the 302,208 CD4 transition observations and generated CD4-based disease progression probability matrix as shown in Table A7. Disease progression for diagnosed and infected individuals who have not been initiated ART was derived from the peer-reviewed literature [[8](#_ENREF_8),[39](#_ENREF_39),[40](#_ENREF_40),[41](#_ENREF_41),[42](#_ENREF_42)] as shown in Table A1.

*2.2 ART and Non-ART Medical Costs*

Cost and QALY parameters were included in Table A8. These costs were either adapted from published literature, Guangxi or China-based population-level studies, or derived directly from Guangxi CDC [[3](#_ENREF_3),[43](#_ENREF_43),[44](#_ENREF_44),[45](#_ENREF_45),[46](#_ENREF_46)]. Unit costs of ART were obtained from Guangxi CDC.

Screening-related costs were composed of the costs of the HIV screening test, HIV confirmatory western blot test for standard of care, behavior counselling, CD4 count test (differed for standard of care and intervention scenario) and pVL (plasma viral load) test for One4All intervention.

Monthly costs of healthcare utilization for the HIV-uninfected population of Guangxi were drawn from *Li et al.* [[3](#_ENREF_3)], and represented male and female residents aged 15-64 years. IDU and MSM/IDU healthcare cost estimates were inflated by a factor of 2.38, consistent with observed differences among HIV-positive IDU and non-IDU [[47](#_ENREF_47)].

*2.3 Incremental Cost-Effectiveness Analysis*

The primary objective was to determine the incremental cost-effectiveness of observed standard of care with ordinary screening procedure in Guangxi Zhuang Autonomous Region compared with One4All screening intervention scenario that significantly shortens screening procedure and improves the rate of screening completeness.

We numerically solved the system on nonlinear differential equations to calculate the number of individuals in each compartment over time. We then calculated a range of outcome measures, including HIV prevalence, the number of new HIV infections, mortality, discounted costs and health benefits (quality-adjusted life years gained), and incremental cost-effectiveness. Total health benefits for the entire population were measured in discounted quality-adjusted life years (QALYs). We executed the analysis to capture health benefits accumulated within the study time horizon (2014-2038).

$QALYs= \int_{0}^{T} e^{-rt}\sum_{\forall i} q_{i}X_{it}dt$

Total discounted costs for the entire population were calculated as the sum of annual healthcare costs for all individuals, including costs of antiretroviral treatment, non-ART heath care and screening-related activities over the specified timeframes:

$C_{Total}=\int_{0}^{T} e^{-rt}\sum_{\forall i} c_{it}X_{it}dt= \int_{0}^{T} e^{-rt}\left[ \sum_{i=S_{1},S_{2}\leq2} C_{neg,i}X_{it}+\sum_{i\neq S_{1},S_{2}>2} C_{pos,it}X_{it} \right]dt$

Where C_neg,i_ represents health care costs accumulated by HIV-negative individuals (including the costs of HIV screening and other health resource use), C_pos,it_ represents the costs of HIV screening, ART and non-ART costs attributable to HIV-positive individuals across health states i and time t. Finally, we calculated the incremental cost-effectiveness ratio (ICER) of the care with boosted intervention screening test versus the standard of care with conventional screening procedure during the study period, as well as alternative scenarios in sensitivity analysis.

$$ICER= \frac{{Cost}_{Intervention}-{Cost}_{SOC}}{{QALY}_{Intervention}-{QALY}_{SOC}}$$

**3.0 Sensitivity Analyses**

We conducted a series of one-way sensitivity analyses to test structural and parameter uncertainty within the model, as well as the sensitivity of the results to the reach and effectiveness of the trial. The results of sensitivity analyses were presented in Table A10 and Figure A7. First, alternate scenarios were constructed to determine the sensitivity of results to parameters estimated with the greatest level of uncertainty, including the proportions of MSM and IDU within the susceptible population, and baseline HIV prevalence, respectively as shown in Figure A7. We re-calibrated our model in each sensitivity analysis to maintain the validity of our model estimates. We note that in the analysis of the scenario with baseline HIV prevalence of 80,000, in accordance with the lower bound of the 2011 UNAIDS report [[27](#_ENREF_27)], we adjusted the values of transmission-related risk behavioural parameters (i.e. the probability of needle sharing and probability of condom use) to calibrate the model. Second, we tested the sensitivity of our model at the lower and upper bounds of the 95% confidence interval on the primary outcome of the One4All trial (odds of HIV test receipt at 30 days) (Figure A7, Panel A). Third, to test the sensitivity of our results to the baseline assumptions of holding epidemiological and health service use indicators (including the MMT index (affecting probability of needle sharing), STD index (affecting probability of condom use), annual screening probability (affecting monthly screening rate) and rate of ART initiation for diagnosed individuals (*α_t_*)) constant at 2014 levels in the projected study time horizon, we fitted the available data from year 2011-2014 with parametric trend lines to generate predictions for 2015-2038. We then conducted sensitivity analyses by incorporating these new estimates individually and jointly. Quadratic curves were chosen on the basis of goodness of fit and appropriateness in growth predictions. The resulting R^2^ and p-values were listed in Table A9. Forth, we considered alternate scenarios whereby the intervention was able to reach a broader population of PLHIV with CD4<350 from all risk groups during routine medical examinations, as presented in Table A10. Finally, we executed an additional sensitivity analysis, varying the odds of HIV test receipt at 30 days to identify thresholds at which the One4All intervention would be deemed cost-inefficient relative to the current standard of care. A complete description of all sensitivity analysis scenarios, including references for alternate estimates, is provided in Table A9.

**Appendix Tables**

**Table A1. Dynamic compartmental model variables**

| **Description** | **Symbol** | **Value** | **Source** |
| --- | --- | --- | --- |
| Number of individuals in susceptible compartment i at time t (initial value) | *S_i_* | 31,832,050 | [[16](#_ENREF_16),[24](#_ENREF_24),[25](#_ENREF_25),[26](#_ENREF_26)] |
| Number of individuals in HIV-infected compartment i at time t (initial value) | *I_i_* | 47,113 | [[4](#_ENREF_4),[27](#_ENREF_27),[28](#_ENREF_28),[29](#_ENREF_29),[30](#_ENREF_30)] |
| Number of individuals in HIV-diagnosed compartment i at time t (initial value) | *D_i_* | 35,252 | Guangxi CDC |
| Number of individuals in HIV treatment compartment i at time t (initial value) | *T_i_* | 14,749 | Guangxi CDC |
| Monthly entry rate of individuals into compartment i (baseline) | *ρ* | 0.001792 | [[16](#_ENREF_16),[20](#_ENREF_20)] |
| Average duration uninfected individuals in compartment i remain ‘identified’ after screening | *1/ω_i_* | 12 months | assumption* |
| Monthly HIV screening rate for individuals in compartment I (low risk) | $\psi_{i}^{l}$ | time-varying | [[21](#_ENREF_21)] |
| Monthly HIV screening rate for individuals in compartment I (high risk) | $\psi_{i}^{h}$ | time-varying | calibrated |
| ‘One4All’ screening intervention multiplier (heterosexual individuals only) |  | 3.45 | One4All, [[23](#_ENREF_23)] |
| Symptom-based monthly case finding rate for infected individuals in compartment i | *ν_i_* |  |  |
| high risk (CD4: <200) |  | 0.00923 | [[3](#_ENREF_3),[8](#_ENREF_8)] |
| low risk (CD4: 200-349) |  | 0.02082 | [[3](#_ENREF_3),[8](#_ENREF_8)] |
| Total sufficient contact rate | *λ_i,j(t)_* | time-varying | calculated |
| Mortality and maturation rate for individuals in compartment i | *μ_i_* |  |  |
| Monthly mortality rate (background) (aged 15-64 years) |  |  |  |
| HETERO or MSM |  | 0.00021 | [[16](#_ENREF_16),[20](#_ENREF_20)] |
| IDU or MSM/IDU |  | 0.00232 | [[3](#_ENREF_3)] |
| Monthly mortality rate (background) (aged 65-95 years)** |  | 0.00326 | [[16](#_ENREF_16),[20](#_ENREF_20)] |
| Monthly mortality rate (HIV infected and diagnosed) |  |  |  |
| HETERO or MSM |  |  | calculated, [[3](#_ENREF_3)] |
| Asymptomatic (CD4: ≥350) |  | 0.00225 |  |
| Symptomatic (CD4: 200-349) |  | 0.00600 |  |
| AIDS (CD4: <200) |  | 0.02120 |  |
| IDU or MSM/IDU |  |  | calculated, [[3](#_ENREF_3)] |
| Asymptomatic (CD4: ≥350) |  | 0.00441 |  |
| Symptomatic (CD4: 200-349) |  | 0.00826 |  |
| AIDS (CD4: <200) |  | 0.02387 |  |
| Monthly mortality rate (on ART) |  | calculated (Table A7) |  |
| HETERO or MSM |  |  | calculated |
| CD4: ≥500 |  | 0.00049 |  |
| CD4: 350-499 |  | 0.00052 |  |
| CD4: 200-349 |  | 0.00063 |  |
| CD4: <200 |  | 0.00268 |  |
| IDU or MSM/IDU |  |  | calculated |
| CD4: ≥500 |  | 0.00261 |  |
| CD4: 350-499 |  | 0.00264 |  |
| CD4: 200-349 |  | 0.00275 |  |
| CD4: <200 |  | 0.00485 |  |
| Monthly maturation rate |  | 0.00058 | [[16](#_ENREF_16),[20](#_ENREF_20)] |
| HIV disease progression rate for individuals not on ART | $\theta_{\overline{T}_{i}}$ |  |  |
| CD4:≥500 to CD4: 350-499 |  | 0.02209 | [[8](#_ENREF_8),[39](#_ENREF_39),[40](#_ENREF_40),[41](#_ENREF_41),[42](#_ENREF_42)] |
| CD4: 350-499 to CD4: 200-349 |  | 0.02209 |  |
| CD4: 200-349 to CD4: <200 |  | 0.02209 |  |
| HIV disease progression rate for individuals on ART in compartment i | $\theta_{T_{it}j(t)}\theta_{T_{it}}$ | calculated (Table A7) |  |
| Rate of individuals from diagnosed compartment i initiating antiretroviral treatment | *α_t_* | time-varying | calibrated |
| Fraction of individuals from compartment I who begin ART at CD4≤350 cells/mm^3^ | *φ_i_* | 0.3 | [[3](#_ENREF_3),[47](#_ENREF_47)] |

** The proportion of HIV positive population aged 65-95 years among HIV positive individuals was estimated to be 15%, consistent to the findings of *Wang et al*  [[21](#_ENREF_21)], and this mortality rate is proportionally added with background morality rate (aged 15-64 years) to estimate 65-95 age group’s background mortality rate for individuals in infectious, diagnosis or on-ART states.

**Table A2. Assumptions regarding HIV contact between HIV-infected and uninfected individuals, by risk group**

|  | MSM | MSM/IDU | IDU | HETERO |
| --- | --- | --- | --- | --- |
| MSM | Homosexual sex | Homosexual sex | Heterosexual sex* | Heterosexual sex* |
| MSM/IDU | Homosexual sex | Needle sharing, Homosexual sex | Needle sharing, Heterosexual sex* | Heterosexual sex* |
| IDU | Heterosexual sex* | Needle sharing, Heterosexual sex* | Needle sharing, Heterosexual sex* | Heterosexual sex* |
| HETERO | Heterosexual sex* | Heterosexual sex* | Heterosexual sex* | Heterosexual sex* |

MSM: Men who have sex with men; IDU: injection drug users; HETERO: heterosexual individuals (non-injection drug users). *To adjust for the proportion of males in IDU and HETERO who were not eligible for heterosexual contact with MSM and MSM/IDU, we multiplied the number of heterosexual partners in these partnerships by 0.5.

**Table A3. HIV transmission parameters**

| **Parameter** | **Symbol** | **Value** | **Source** |
| --- | --- | --- | --- |
| ***Needle Sharing Parameters*** |  |  |  |
| Number of injections* (monthly) | d_t_ | 16.67 | [[3](#_ENREF_3),[44](#_ENREF_44),[48](#_ENREF_48)] |
| Probability of shared injection | s | 0.264 | [[4](#_ENREF_4)] |
| Probability of transmission per shared injection: CD4: ≥500 | $\tau_{1}$ | 0.002 | [[3](#_ENREF_3),[39](#_ENREF_39),[49](#_ENREF_49),[50](#_ENREF_50)] |
| Probability of transmission per shared injection: CD4: 350-499 | $\tau_{2}$ | 0.002 | [[3](#_ENREF_3),[39](#_ENREF_39),[49](#_ENREF_49),[50](#_ENREF_50)] |
| Probability of transmission per shared injection: CD4: 200-349 | $\tau_{3}$ | 0.003 | [[3](#_ENREF_3),[39](#_ENREF_39),[49](#_ENREF_49),[50](#_ENREF_50)] |
| Probability of transmission per shared injection: CD4: <200 | $\tau_{4}$ | 0.003 | [[3](#_ENREF_3),[39](#_ENREF_39),[49](#_ENREF_49),[50](#_ENREF_50)] |
| Reduced probability of transmission on ART | $\delta_{I}$ | 0.5 | [[8](#_ENREF_8),[44](#_ENREF_44)] |
| ***Homosexual Sex Parameters*** |  |  |  |
| No sexual partners (annual) | n^s^ | 5 | [[7](#_ENREF_7)] |
| Condom use probability | $u_{it}^{s}$ | 0.464 | [[4](#_ENREF_4)] |
| Probability of transmission: CD4: ≥500 | $\sigma_{M1}$ | 0.04 | [[3](#_ENREF_3),[51](#_ENREF_51),[52](#_ENREF_52),[53](#_ENREF_53),[54](#_ENREF_54)] |
| Probability of transmission: CD4: 350-499 | $\sigma_{M2}$ | 0.05 | [[3](#_ENREF_3),[51](#_ENREF_51),[52](#_ENREF_52),[53](#_ENREF_53),[54](#_ENREF_54)] |
| Probability of transmission: CD4: 200-349 | $\sigma_{M3}$ | 0.05 | [[3](#_ENREF_3),[51](#_ENREF_51),[52](#_ENREF_52),[53](#_ENREF_53),[54](#_ENREF_54)] |
| Probability of transmission: CD4: <200 | $\sigma_{M4}$ | 0.12 | [[3](#_ENREF_3),[51](#_ENREF_51),[52](#_ENREF_52),[53](#_ENREF_53),[54](#_ENREF_54)] |
| Reduced probability of transmission on ART | $\delta_{M}$ | 0.90 | [[3](#_ENREF_3),[51](#_ENREF_51),[52](#_ENREF_52),[53](#_ENREF_53),[54](#_ENREF_54)] |
| Reduced probability of sexual contact due to diagnosis for PLHIV individuals | $\varepsilon_{S\_M1}$ | 0.20 | [[3](#_ENREF_3),[9](#_ENREF_9)] |
| Reduced probability of sexual contact due to diagnosis for AIDS individuals | $\varepsilon_{S\_M2}$ | 0.90 | [[3](#_ENREF_3)] |
| Condom effectiveness | $\kappa$ | 0.8 | [[5](#_ENREF_5)] |
| ***Heterosexual Sex Parameters*** |  |  |  |
| No sexual partners: MSM | n^o,M^ | 0.3 | [[55](#_ENREF_55)] |
| No sexual partners: MSM/IDU | n^o,MI^ | 0.3 | [[55](#_ENREF_55)] |
| No sexual partners: IDU | n^o,I^ | 3 | [[48](#_ENREF_48)] |
| No sexual partners: HETERO | n^o,H^ | 1.2 | [[30](#_ENREF_30)] |
| Condom use probability: MSM | $u_{it}^{o,M}$ | 0.295 | [[5](#_ENREF_5)] |
| Condom use probability: MSM/IDU | $u_{it}^{o,MI}$ | 0.295 | [[5](#_ENREF_5),[6](#_ENREF_6)] |
| Condom use probability: IDU | $u_{it}^{o,I}$ | 0.383 | [[6](#_ENREF_6)] |
| Condom use probability: HETERO | $u_{it}^{o,H}$ | 0.11 | [[7](#_ENREF_7)] |
| Probability of transmission: CD4: ≥500 | $\sigma_{H1}$ | 0.03 | [[3](#_ENREF_3),[13](#_ENREF_13),[56](#_ENREF_56),[57](#_ENREF_57),[58](#_ENREF_58),[59](#_ENREF_59),[60](#_ENREF_60)] |
| Probability of transmission: CD4: 350-499 | $\sigma_{H2}$ | 0.03 | [[3](#_ENREF_3),[13](#_ENREF_13),[56](#_ENREF_56),[57](#_ENREF_57),[58](#_ENREF_58),[59](#_ENREF_59),[60](#_ENREF_60)] |
| Probability of transmission: CD4: 200-349 | $\sigma_{H3}$ | 0.04 | [[3](#_ENREF_3),[13](#_ENREF_13),[56](#_ENREF_56),[57](#_ENREF_57),[58](#_ENREF_58),[59](#_ENREF_59),[60](#_ENREF_60)] |
| Probability of transmission: CD4: <200 | $\sigma_{H4}$ | 0.08 | [[3](#_ENREF_3),[13](#_ENREF_13),[56](#_ENREF_56),[57](#_ENREF_57),[58](#_ENREF_58),[59](#_ENREF_59),[60](#_ENREF_60)] |
| Reduced probability of sexual contact due to diagnosis for PLHIV individuals | $\varepsilon_{S\_H1}$ | 0.20 | [[3](#_ENREF_3),[9](#_ENREF_9)] |
| Reduced probability of sexual contact due to diagnosis for AIDS individuals | $\varepsilon_{S\_H2}$ | 0.90 | [[3](#_ENREF_3)] |
| Reduced probability of transmission on ART | $\delta_{H}$ | 0.90 | [[3](#_ENREF_3),[51](#_ENREF_51),[52](#_ENREF_52),[53](#_ENREF_53),[54](#_ENREF_54)] |
| Condom effectiveness | $\kappa$ | 0.80 | [[61](#_ENREF_61),[62](#_ENREF_62),[63](#_ENREF_63)] |

**Table A4. Complete starting values across risk groups and HIV disease progression states: Guangxi, 12/2010**

|  |  | MSM | IDU | MSM/IDU | HETERO | Total |
| --- | --- | --- | --- | --- | --- | --- |
| Infected | CD4: ≥500 | 1461 | 2117 | 312 | 4115 | 8005 |
|  | CD4: 350-499 | 1844 | 2671 | 393 | 5192 | 10100 |
|  | CD4: 200-349 | 2772 | 4017 | 591 | 7806 | 15186 |
|  | CD4: <200 | 2678 | 3880 | 571 | 7541 | 14671 |
| Diagnosed | CD4: ≥500 | 806 | 1967 | 179 | 2932 | 5884 |
|  | CD4: 350-499 | 1016 | 2482 | 226 | 3700 | 7424 |
|  | CD4: 200-349 | 1528 | 3732 | 339 | 5563 | 11161 |
|  | CD4: <200 | 1476 | 3605 | 328 | 5374 | 10783 |
| On ART | CD4: ≥500 | 53 | 428 | 4 | 1977 | 2462 |
|  | CD4: 350-499 | 66 | 540 | 5 | 2495 | 3106 |
|  | CD4: 200-349 | 100 | 811 | 8 | 3751 | 4670 |
|  | CD4: <200 | 96 | 784 | 8 | 3623 | 4511 |
|  | Total | 13897 | 27034 | 2963 | 54068 | 97962 |

MSM: Men who have sex with men; IDU: injection drug users; HETERO: heterosexual individuals

**Table A5. Baseline characteristics of the population on ART (42,267 patients): Guangxi, 12/2014**

| **Baseline Characteristics** | |
| --- | --- |
| Number of individuals | 42267 |
| Number of observations | 344475 |
| Number of transitions | 302,208 |
| Follow-up in years, median (IQR) (year) | 2.68 (1.38, 4.43) |
| Number of CD4 records per person, median (IQR) | 9 (5, 12) |
| Baseline CD4 (cell count/mm^3^), N (%) |  |
| ≥500 | 937(2.22%) |
| 350-499 | 2931(6.93%) |
| 200-349 | 12599(29.81%) |
| <200 | 25800(62.04%) |
| Males, N (%) | 27529（65.13%） |
| Age at ART initiation, median (IQR) | 41.8 (32.5,56.2) |
| Year of first ARV, N (%) |  |
| 2003-2010 | 14635 (34.63%) |
| 2011-2014 | 27632 (65.37%) |
| Transmission route, N (%) |  |
| Blood (plasma) donation | 105(0.25%) |
| IDU | 4072(9.75%) |
| Male to male sexual contact | 491(1.18%) |
| Heterosexual contact | 36021(86.24%) |
| Unclear | 1078(2.57%) |

**Table A6. Number of transitions between CD4-based strata for PLHIV on ART (302,208 observations)**

| CD4 Stratum at Time t | CD4 Stratum at Time t+1 | | | | | |
| --- | --- | --- | --- | --- | --- | --- |
|  | ≥500 | 350-499 | 200-349 | <200 | Off Therapy | Death |
| ≥500 | 33052 | 10519 | 2299 | 461 | 694 | 118 |
| 350-499 | 16542 | 28457 | 12335 | 1068 | 911 | 167 |
| 200-349 | 6073 | 23219 | 48945 | 9304 | 1549 | 339 |
| <200 | 1538 | 3895 | 23288 | 55101 | 1717 | 857 |
| Off Therapy | 467 | 513 | 735 | 1120 | 16922 | 3 |

**Table A7. CD4-based HIV disease progression probability matrix for PLHIV on ART**

| CD4 Stratum at Time t | CD4 Stratum at Time t+1 | | | | | |
| --- | --- | --- | --- | --- | --- | --- |
|  | ≥500 | 350-499 | 200-349 | <200 | Off Therapy | Death |
| ≥500 | 0.91395 | 0.07808 | 0.00379 | 0.00009 | 0.00361 | 0.00049 |
| 350-499 | 0.09826 | 0.81572 | 0.08064 | 0.00167 | 0.00320 | 0.00052 |
| 200-349 | 0.00555 | 0.09425 | 0.86068 | 0.03497 | 0.00392 | 0.00063 |
| <200 | 0.00020 | 0.00469 | 0.08511 | 0.90301 | 0.00431 | 0.00268 |
| Off Therapy | 0.00769 | 0.00816 | 0.012033 | 0.00186 | 0.95345 | 0.00003 |

**Table A8. Costs and quality-adjusted life year estimates**

| ***Costs (2014 CNY)*** | **Value** | **Source** |
| --- | --- | --- |
| ART costs^^ (monthly): | 221 | Guangxi CDC |
| Non-ART medical costs^#^: IDU | monthly |  |
| CD4: ≥500 | 4830 | [[39](#_ENREF_39),[46](#_ENREF_46)] |
| CD4: 350-499 | 4830 | [[39](#_ENREF_39),[46](#_ENREF_46)] |
| CD4: 200-349 (untreated) | 7974 | [[39](#_ENREF_39),[46](#_ENREF_46)] |
| CD4: 200-349 (treated) | 7131 | [[39](#_ENREF_39),[46](#_ENREF_46)] |
| CD4: <200 (untreated) | 10689 | [[39](#_ENREF_39),[46](#_ENREF_46)] |
| CD4: <200 (treated) | 9321 | [[39](#_ENREF_39),[46](#_ENREF_46)] |
| Non-ART medical costs^#^: non-IDU | monthly |  |
| CD4: ≥500 | 2029 | [[3](#_ENREF_3),[43](#_ENREF_43)] |
| CD4: 350-499 | 2029 | [[3](#_ENREF_3),[43](#_ENREF_43)] |
| CD4: 200-349 (untreated) | 3350 | [[3](#_ENREF_3),[43](#_ENREF_43)] |
| CD4: 200-349 (treated) | 2996 | [[3](#_ENREF_3),[43](#_ENREF_43)] |
| CD4: <200 (untreated) | 4491 | [[3](#_ENREF_3),[43](#_ENREF_43)] |
| CD4: <200 (treated) | 3916 | [[3](#_ENREF_3),[43](#_ENREF_43)] |
| HIV Screening-related costs |  |  |
| Standard of care- Total screening cost for positive screen^ | 846 |  |
| HIV ELISA antibody test | 24 | Guangxi CDC |
| Confirmatory western blot test | 500 | Guangxi CDC |
| Behaviour counselling | 82 | Guangxi CDC |
| CD4 count test | 240 | [[3](#_ENREF_3),[64](#_ENREF_64)] |
| One4All Intervention – Total screening cost for positive screen^ | 2182 |  |
| POC^##^ HIV screening test | 24 | Guangxi CDC |
| POC^##^ CD4 count test | 576 | Guangxi CDC, [[65](#_ENREF_65),[66](#_ENREF_66)] |
| Behaviour counselling | 82 | [[3](#_ENREF_3),[64](#_ENREF_64)] |
| Viral load test | 1500 | Guangxi CDC |
| Monthly cost of ART | 221 | Guangxi CDC |
| Medical care costs^#^: HIV-negative, MSM | 78 | [[3](#_ENREF_3),[39](#_ENREF_39),[46](#_ENREF_46)] |
| Medical care costs^#^: HIV-negative, IDU | 196 | [[3](#_ENREF_3),[39](#_ENREF_39),[46](#_ENREF_46)] |
| Medical care costs^#^: HIV-negative, HETERO | 82 | [[3](#_ENREF_3),[39](#_ENREF_39),[46](#_ENREF_46)] |
| ***Quality-adjusted life years*** |  |  |
| Susceptible | 1.00 |  |
| Infected: CD4: ≥500 | 0.90 | [[3](#_ENREF_3),[8](#_ENREF_8),[67](#_ENREF_67),[68](#_ENREF_68),[69](#_ENREF_69),[70](#_ENREF_70)] |
| Infected: CD4: 350-499 | 0.79 | [[3](#_ENREF_3),[8](#_ENREF_8),[67](#_ENREF_67),[68](#_ENREF_68),[69](#_ENREF_69),[70](#_ENREF_70)] |
| Infected: CD4: 200-349 | 0.79 | [[3](#_ENREF_3),[8](#_ENREF_8),[67](#_ENREF_67),[68](#_ENREF_68),[69](#_ENREF_69),[70](#_ENREF_70)] |
| Infected: CD4: <200 | 0.72 | [[3](#_ENREF_3),[8](#_ENREF_8),[67](#_ENREF_67),[68](#_ENREF_68),[69](#_ENREF_69),[70](#_ENREF_70)] |
| Diagnosed: CD4: ≥500 | 0.85 | [[3](#_ENREF_3),[8](#_ENREF_8),[67](#_ENREF_67),[68](#_ENREF_68),[69](#_ENREF_69),[70](#_ENREF_70)] |
| Diagnosed: CD4: 350-499 | 0.72 | [[3](#_ENREF_3),[8](#_ENREF_8),[67](#_ENREF_67),[68](#_ENREF_68),[69](#_ENREF_69),[70](#_ENREF_70)] |
| Diagnosed: CD4: 200-349 | 0.72 | [[3](#_ENREF_3),[8](#_ENREF_8),[67](#_ENREF_67),[68](#_ENREF_68),[69](#_ENREF_69),[70](#_ENREF_70)] |
| Diagnosed: CD4: <200 | 0.68 | [[3](#_ENREF_3),[8](#_ENREF_8),[67](#_ENREF_67),[68](#_ENREF_68),[69](#_ENREF_69),[70](#_ENREF_70)] |
| On ART: CD4: ≥500 | 0.85 | [[3](#_ENREF_3),[8](#_ENREF_8),[67](#_ENREF_67),[68](#_ENREF_68),[69](#_ENREF_69),[70](#_ENREF_70)] |
| On ART: CD4: 350-499 | 0.83 | [[3](#_ENREF_3),[8](#_ENREF_8),[67](#_ENREF_67),[68](#_ENREF_68),[69](#_ENREF_69),[70](#_ENREF_70)] |
| On ART: CD4: 200-349 | 0.83 | [[3](#_ENREF_3),[8](#_ENREF_8),[67](#_ENREF_67),[68](#_ENREF_68),[69](#_ENREF_69),[70](#_ENREF_70)] |
| On ART: CD4: <200 | 0.82 | [[3](#_ENREF_3),[8](#_ENREF_8),[67](#_ENREF_67),[68](#_ENREF_68),[69](#_ENREF_69),[70](#_ENREF_70)] |
| IDU multiplier | 0.9 | [[39](#_ENREF_39),[50](#_ENREF_50),[71](#_ENREF_71)] |

IDU: injection drug users; MSM: men who have sex with men; HETERO: heterosexuals; ART: highly active antiretroviral treatment * Transitions over a month were estimated from any of the four CD4-based health states to any of the CD4-based states i≠j. ** Calculated values; see supplementary appendix for complete description. ^ Screening costs of those screening negative include only the HIV ELISA antibody test (SOC) or the POC HIV screening test (One4All). ^^ includes ART medication costs and associated pharmacy dispensation costs. ^#^ Includes non-ART medication costs and associated pharmacy dispensation costs, costs of physician billings for outpatient care and hospitalization cost. ## POC stands for ‘point-of-care’ in the proposed One4All screening intervention that CD4 cell count test will be conducted at local healthcare facilities instead of county or city CDC lab.

**Table A9. Sensitivity analysis description**

| **Scenario** | | | **Baseline** | **Note** |
| --- | --- | --- | --- | --- |
| Baseline Susceptible Population  (SP) | Adjusting proportion of MSM among susceptible population; diagnosed and on-ART population remained unchanged | 1. Proportion of MSM among SP = 2% 2. Proportion of MSM among SP = 1% | MSM among SP: 1.116% (including 8% MSM/IDU) | Reference: [[25](#_ENREF_25)] |
|  | Adjusting number of IDU among SP; diagnosed and on-ART population remained unchanged | 1. Number of IDU among SP=360,736 2. Number of IDU among SP=226,034 | Number of IDU among SP = 293,551 (excluding MSM/IDU) | Scenario 3 was based on news report [[72](#_ENREF_72)] and extrapolations; Scenario 4 was assumed as a comparison |
| Baseline Infected Population  (IP) | Adjusting total infected population; susceptible population, diagnosed and on-ART population remained unchanged | 1. Number of IP=80,000 2. Number of IP=100,000 | Total number of IP=97,114 | Reference: [[27](#_ENREF_27)] |
| Intervention Effectiveness | Odds ratio (95% CI*) | 1. Upper bound: 124.46   Relative risk^#^: 3.82   1. Lower bound: 3.81   Relative risk^#^: 2.22 | Odds ratio: 21.78  Relative risk^#^: 3.45 | CTN-0056 intervention trial primary outcome |
|  | Odds ratio | 1. Odds Ratio: (1,∞)   Relative risk^#^: (1, 4) |  |  |
| Projected change in key epidemiological and health service use indicators | STD index | 1. Fitted with quadratic curve | Constant since 2015 | R square=0.992  p-value=0.004 |
|  | MMT index | 1. Fitted with quadratic curve | Constant since 2015 | R square=0.425  p-value=0.348 |
|  | Screening probability | 1. Fitted with quadratic curve | Constant since 2015 | R square=0.988  p-value=0.006 |
|  | ART initiation rate | 1. Fitted with quadratic curve | Constant since 2015 | R square=0.949  p-value=0.026 |
|  | Scenario 10-13 combined | 1. Fitted with quadratic curve | Constant since 2015 |  |
| Intervention Expansion | Expanding ‘One4All’ screening intervention to all four risk groups (CD4<350) | 1. To all risk groups | Intervention to heterosexual group (CD4<350) only | Presented in Table A11 |

* CI stands for confidence interval

# Relative risk derived from odds ratio^23^ and used to as multiplier in calculating monthly screening rate

**Table A10. Sensitivity analysis on One4All screening intervention expansion**

|  | **Incident cases** | **Deaths among PLHIV** | **ART costs (PLHIV)** | **Non-ART costs (PLHIV)** | **Total costs** | **QALYs** | **ICER** |
| --- | --- | --- | --- | --- | --- | --- | --- |
|  |  |  | 2014 Million CNY | 2014 Million CNY | 2014 Million CNY | Million | 2014 CNY |
| ***1-year time horizon*** | |  |  |  |  |  |  |
| Standard of care | 5,944 | 5,285 | 102.458 | 4,921.04 | 37,682.24 | 32.640 |  |
| One4All_All | 5,806 | 5,203 | 104.325 | 4,921.86 | 37,685.00 | 32.640 | 91,970 |
| ***5-year time horizon*** | | | | | | | |
| Standard of care | 28,184 | 20,582 | 577.914 | 22,705.76 | 179,643.60 | 156.272 |  |
| One4All_All | 27,515 | 19,823 | 593.927 | 22,652.18 | 179,607.98 | 156.274 | Dominant |
| ***25-year time horizon*** | | | | | | | |
| Standard of care | 171,923 | 103,519 | 2,889.322 | 99,970.77 | 738,246.57 | 635.184 |  |
| One4All_All | 169,578 | 101,075 | 2,948.512 | 99,930.02 | 738,291.06 | 635.210 | 1,690 |

Year 2014, in CNY:

GDP per capita = 46,629

3*GDP per capita = 139,887

**Appendix Figures**

**Figure A1. Model diagram**

*I*_1_: Infected

*D*_1_: Diagnosed

*S*_1_: Susceptible

Not screened

CD4≥500

CD4≥500

*T*_1_: On ART

CD4≥500

*I*_2_: Infected

*D*_2_: Diagnosed

*T*_2_: On ART

*S*_2_: Susceptible

Screened

CD4:350-499

CD4:350-499

CD4:350-499

*I*_3_: Infected

*D*_3_: Diagnosed

*T*_3_: On ART

CD4: 200-349

CD4: 200-349

CD4: 200-349

*I*_4_: Infected

*D*_4_: Diagnosed

*T*_4_: On ART

CD4<200

CD4<200

CD4<200

$$\sum_{j\neq S_{1},S_{2}} \lambda_{S_{2},j}$$

$$\sum_{j\neq S_{1},S_{2}} \lambda_{S_{1},j}$$

$$a_{t}$$

$$a_{t}$$

$$\rho$$

$$\psi$$

$$\omega$$

$$\psi_{1}$$

$$\psi_{2}$$

$$(\psi_{3}+\nu_{3})(1-\varphi)$$

$$(\psi_{4}+\nu_{4}) (1-\varphi)$$

$$\theta_{\bar{T}_{1}}$$

$$\theta_{\bar{T}_{2}}$$

$$\theta_{\bar{T}_{3}}$$

$$\theta_{\bar{T}_{1}}$$

$$\theta_{\bar{T}_{3}}$$

$$\theta_{\bar{T}_{2}}$$

$$\theta_{T_{1},D_{1}}$$

$$\theta_{T_{2},D_{2}}$$

$$\theta_{T_{3},D_{3}}$$

$$\theta_{T_{4},D_{4}}$$

$$\theta_{T_{i},T_{j}}$$

$$\theta_{T_{i},T_{j}}$$

$$\theta_{T_{i},T_{j}}$$

$$(\psi_{4}+\nu_{4}) \varphi$$

$$(\psi_{3}+\nu_{3})\varphi$$

**Figure A2. Dynamic compartmental model ‘trace’: number of individuals in infected, diagnosed and on-ART in observed standard of care and One4All screening intervention scenario**

**PANEL A: All risk group**

**PANEL B: Heterosexual group**

**Figure A3. Parameters used to proxy changes in HIV risk behavior**

**PANEL A: MMT initiation index**

**PANEL B: Resulting time-dependent mean monthly number of shared injections***

* Inverse of the MMT initiation rate multiplied by the fixed baseline number of injections per year

**PANEL C: Non-HIV sexually transmitted disease (STD) rate**

**PANEL D: Resulting time-dependent probability of condom use, by partnership**

**Figure A4. Time-dependent parameter estimates: observed and derived annual rate of HIV screening**

**Figure A5. Model calibration**

**PANEL A: Observed total new diagnosis**

**PANEL B: Observed ART scale-up**

**Figure A6. Results of model validation**

**PANEL A: Mortality of all causes among diagnosed population**

**PANEL B: Population aged 15-64 in Guangxi**

**Figure A7. Results of sensitivity analysis - tornado diagram**

Baseline model:

(1) 97114 total PLHIV at baseline;

(2) Intervention effectiveness, OR=21.78;

(3) 293551 susceptible IDUs;

(4) MSM: 1.116% among susceptible population;

(5) Constant estimates for STD index, MMT index, annual screening probability and annual ART initiation rate since year 2015.

References

1. China free antiretroviral treatment manual (2012). Beijing: Ministry of Health Working Group on Clinical AIDS Treatment.

2. NCAIDS N, China CDC (2011-2014) Update on the AIDS/STD epidemic in China and main response in control and prevention. Chinese Journal of AIDS&STD.

3. Li J, Gilmour S, Zhang H, Koyanagi A, Shibuya K (2012) The epidemiological impact and cost-effectiveness of HIV testing, antiretroviral treatment and harm reduction programs. AIDS 26: 2069-2078.

4. Chen Y, Chen J, Liu W, Xu G, Wang H, et al. (2012) Analysis of high risk behaviors of drug users related to HIV/AIDS transmitting in some area of Guangxi. Chinese Journal of AIDS & STD 18: 86-88.

5. She Y, Zhong X, Zhang Y, Hao B, Liang H, et al. (2010) Knowledge and Behaviors Related to HIV/AIDS among Men who Have Sex with Men in Western China. Journal of Chongqing Medical University 135: 1902-1905.

6. Chen Y, Tang Z, Shen Z, Zhu Q, Liang F, et al. (2013) Trend of HIV/AIDS Epidemic among Drug User in Guangxi Zhuang Autonomous Region, 2007-2012. Disease Surveillance 28: 643-647.

7. Wang S, Moss JR, Hiller JE (2011) The cost-effectiveness of HIV voluntary counseling and testing in China. Asia-Pacific journal of public health / Asia-Pacific Academic Consortium for Public Health 23: 620-633.

8. Sanders GD, Bayoumi AM, Sundaram V, Bilir SP, Neukermans CP, et al. (2005 ) Cost-effectiveness of screening for HIV in the era of highly active antiretroviral therapy. N Engl J Med 352: 570-585.

9. Kamb ML, Fishbein M, Douglas JM Jr, Rhodes F, Rogers J, et al. (1998) Efficacy of risk-reduction counseling to prevent human immunodeficiency virus and sexually transmitted diseases: a randomized controlled trial. JAMA 280: 1161-1167.

10. (1998) The NIMH Multisite HIV Prevention Trial: reducing HIV sexual risk behavior.The National Institute of Mental Health (NIMH) Multisite HIV Prevention Trial Group. Science 280: 1889-1894.

11. Cleary PD, Van Devanter N, Rogers TF, Singer E, Shipton-Levy R, et al. (1991) Behavior changes after notification of HIV infection. Am J Public Health 81: 1586-1590.

12. Higgins DL, Galavotti C, O'Reilly KR, Schnell DJ, Moore M, et al. (1991) Evidence for the effects of HIV antibody counseling and testing on risk behaviors. JAMA 226: 2419-2429.

13. Quinn TC, Wawer MJ, Sewankambo N, Serwadda D, Li C, et al. (2000 ) Viral load and heterosexual transmission of human immunodeficiency virus type 1. Rakai Project Study Group. N Engl J Med 342: 921-929.

14. Castilla J, Del Romero J, Hernando V, Marincovich B, Garcia S, et al. (2005) Effectiveness of highly active antiretroviral therapy in reducing heterosexual transmission of HIV. Journal of acquired immune deficiency syndromes 40: 96-101.

15. Porco TC, Martin JN, Page-Shafer KA, Cheng A, Charlebois E, et al. (2004) Decline in HIV infectivity following the introduction of highly active antiretroviral therapy. AIDS 18: 81–88.

16. Region SBoGZA (2015) Guangxi Statistical Yearbook-2015. China Statistics Press.

17. Yang H, Li X, Stanton B, Liu H, Wang N, et al. (2005) Heterosexual transmission of HIV in China: a systematic review of behavioral studies in the past two decades. Sexually transmitted diseases 32: 270-280.

18. Zhang L, Chow EP, Jing J, Zhuang X, Li X, et al. (2013) HIV prevalence in China: integration of surveillance data and a systematic review. The Lancet Infectious diseases 13: 955-963.

19. Wu Z, Rou K, Cui H (2004) The HIV/AIDS epidemic in China: history, current strategies and future challenges. AIDS education and prevention : official publication of the International Society for AIDS Education 16: 7-17.

20. (2012) Tabulation On The 2010 Population Census Of Guangxi Zhuang Autonomous Region. Guangxi Zhuang Autonomous Region Bureau of Statistics, Guangxi Zhuang Autonomous Region Population Census Office.

21. Wang X, Ge X, Tang Z, Shen Z, Lan W, et al. (2015) Epidemiological Characteristics of HIV/AIDS in Guangxi during 2008-2013. J Applied Prev Med 21: 171-222.

22. Centers for Disease Control and Prevention (2008) Persons tested for HIV--United States, 2006. MMWR 57: 845-849.

23. Zhang J, Yu KF (1998) What's the relative risk? A method of correcting the odds ratio in cohort studies of common outcomes. JAMA 280: 1690-1691.

24. Mo Y (2010) Researching the drug problems in Guangxi's frontier district: Guangxi University.

25. Xu Y (2008) Research of psychological and physiological characteristics on male homosexuality: Zhejiang University.

26. Chow EP, Lau JT, Zhuang X, Zhang X, Wang Y, et al. (2014) HIV prevalence trends, risky behaviours, and governmental and community responses to the epidemic among men who have sex with men in China. BioMed research international 2014: 607261.

27. (2011) 2011 Estimates for the HIV/AIDS Epidemic in China. Beijing, China. Ministry of Health, People's Republic of China, United Nations and WHO Programme on HIV/AIDS.

28. Wang X, Lan G, Shen Z, Vermund SH, Zhu Q, et al. (2014) HIV and syphilis prevalence trends among men who have sex with men in Guangxi, China: yearly cross-sectional surveys, 2008-2012. BMC infectious diseases 14: 367.

29. (2016) HIV and AIDS Data Hub for Asia-Pacific. [www.aidsdatahub.org](http://www.aidsdatahub.org).

30. Chen L (2014) Study on HIV/AIDS epidemic characteristics and mathematical discriminative modle of regional categories in Guangxi: Guangxi Medical University.

31. Weinstein MC, O'Brien B, Hornberger J, Jackson J, Johannesson M, et al. ( 2003) Principles of good practice for decision analytic modeling in health-care evaluation: report of the ISPOR Task Force on Good Research Practices--Modeling Studies. Value Health 6: 9-17.

32. Harawa NT, Greenland S, Bingham TA, Johnson DF, Cochran SD, et al. (2004) Associations of race/ethnicity with HIV prevalence and HIV-related behaviors among young men who have sex with men in 7 urban centers in the United States. J Acquir Immune Defic Syndr 35: 526-536.

33. Guihenneuc-Jouyaux C, Richardson S, Longini IM Jr ( 2000) Modeling markers of disease progression by a hidden Markov process: application to characterizing CD4 cell decline. Biometrics 56: 733-741.

34. Sypsa V, Touloumi G, Kenward M, Karafoulidou A, Hatzakis A (2001 ) Comparison of smoothing techniques for CD4 data in a Markov model with states defined by CD4: an example on the estimation of the HIV incubation time distribution. Stat Med 20: 3667-3676.

35. Mathieu E, Loup P, Dellamonica P, Daures JP ( 2005 ) Markov modeling of immunological and virological states in HIV-1 infected patients. Biom J 47: 834-846.

36. Charitos T, de Waal PR, van der Gaag LC ( 2008) Computing short-interval transition matrices of a discrete-time Markov chain from partially observed data. Stat Med 27: 905-921.

37. Satten GA, Longini IM (1996) Markov chains with measurement error: estimating the ‘true’ course of of a marker of the progression of thei human immunodeficiency virus disease. Applied Statistics 45: 275-309.

38. Craig BA, Sendi PP (2002) Estimation of the transition matrix of a discrete-time Markov chain. . Health economics 11: 33-42.

39. Nosyk B, Min JE, Lima VD, Hogg RS, Montaner JS (2015) Cost-effectiveness of population-level expansion of highly active antiretroviral treatment for HIV in British Columbia, Canada: a modelling study. The lancet HIV 2: e393-400.

40. Mellors JW, Muñoz A, Giorgi JV, Margolick JB, Tassoni CJ, et al. (1997) Plasma viral load and CD4+ lymphocytes as prognostic markers of HIV-1 infection. . Ann Intern Med 126: 946-954.

41. Vlahov D, NM G, Hoover D, Flynn C, Bartlett JG, et al. (1998) Prognostic indicators for AIDS and infectious disease death in HIV-infected injection drug users: plasma viral load CD4+ cell count. JAMA 279: 35-40.

42. Hughes MD, Johnson VA, Hirsch MS, Bremer JW, Elbeik T, et al. (1997) Monitoring plasma HIV-1 RNA levels in addition to CD4+ lymphocyte count improves assessment of antiretroviral therapeutic response. Ann Intern Med 126: 929-938.

43. Yang HM, Li J, Wu ZY, Xu LZ, Wang KA (2003) [Study on the utilization of health services and costs of hospital-based medical care for 29 patients with HIV/AIDS in China]. Zhonghua liu xing bing xue za zhi = Zhonghua liuxingbingxue zazhi 24: 393-396.

44. Long EF, Brandeau ML, Owens DK (2010 ) The cost-effectiveness and population outcomes of expanded HIV screening and antiretroviral treatment in the United States. Ann Intern Med 153: 778-789.

45. Bendavid E, Young SD, Katzenstein DA, Bayoumi Am, Sanders GD, et al. (2008) Cost-effectiveness of HIV monitoring strategies in resource-limited settings. Arch Intern Med 168: 1910-1918.

46. Nosyk B, Lima V, Colley G, Yip B, Hogg RS, et al. (2014) Costs of health resource utilization among HIV-positive individuals in British Columbia, Canada: Results from a population-level study. PharmacoEconomics.

47. (2010) China 2010 UNGASS Country Progress Report (2008 - 2009). Ministry of Health of the People’s Republic of China.

48. Wei L, Chen J, Rodolph M, Beauchamp G, Masse B, et al. (2006) HIV incidence, retention, and changes of high-risk behaviors among rural injection drug users in Guangxi, China. Substance abuse 27: 53-61.

49. Homer JB, Clair CLS (1991) A model of HIV transmission through needle sharing. Interfaces 21: 26-49.

50. Zaric GS, Barnett PG, Brandeau ML ( 2000 ) HIV transmission and the cost-effectiveness of methadone maintenance. Am J Public Health 90: 1100-1111.

51. Mastro TD, de Vincenzi I (1996) Probabilities of sexual HIV-1 transmission. AIDS 10: 75-82.

52. Caceres CF, van Griensven GJ (1994) Male homosexual transmission of HIV-1. AIDS 8: 1051-1061.

53. Jacquez JA, Koopman JS, Simon CP, Longini IM Jr. (1994) Role of the primary infection in epidemics of HIV infection in gay cohorts. J Acquir Immune Defic Syndr 7: 1169-1184.

54. Vittinghoff E, Douglas J, Judson F, McKirnan D, MacQueen K, et al. (1999) Per-contact risk of human immunodeficiency virus transmission between male sexual partners. Am J Epidemiol 7: 306-311.

55. Wu Z, Xu J, Liu E, Mao Y, Xiao Y, et al. (2013) HIV and syphilis prevalence among men who have sex with men: a cross-sectional survey of 61 cities in China. Clinical infectious diseases : an official publication of the Infectious Diseases Society of America 57: 298-309.

56. Abbas UL, Anderson RM, Mellors JW (2006) Potential impact of antiretroviral therapy on HIV-1 transmission and AIDS mortality in resource-limited settings. J Acquir Immune Defic Syndr 41: 632-641.

57. Downs AM, De Vincenzi I (1996) Probability of heterosexual transmission of HIV: relationship to the number of unprotected sexual contacts. European Study Group in Heterosexual Transmission of HIV. J Acquir Immune Defic Syndr Hum Retrovirol 11: 388-395.

58. Hollingsworth TD, Anderson RM, Fraser C (2008) HIV-1 transmission, by stage of infection. J Infect Dis 198: 687-693.

59. Padian NS, Shiboski SC, Glass SO, Vittinghoff E (1997) Heterosexual transmission of human immunodeficiency virus (HIV) in northern California: results from a ten-year study. American journal of epidemiology 146: 350-357.

60. Wawer MJ, Gray RH, Sewankambo NK, Serwadda D, Li X, et al. (2005) Rates of HIV-1 transmission per coital act, by stage of HIV-1 infection, in Rakai, Uganda. The Journal of infectious diseases 191: 1403-1409.

61. Davis KR, Weller SC (1999) The effectiveness of condoms in reducing heterosexual transmission of HIV. Family planning perspectives 31: 272-279.

62. Giannou FK, Tsiara CG, Nikolopoulos GK, Talias M, Benetou V, et al. (2015) Condom effectiveness in reducing heterosexual HIV transmission: a systematic review and meta-analysis of studies on HIV serodiscordant couples. Expert review of pharmacoeconomics & outcomes research: 1-11.

63. Cayley WE, Jr. (2004) Effectiveness of condoms in reducing heterosexual transmission of HIV. American family physician 70: 1268-1269.

64. Cheng G, Qian ZH, Hu J (2009) [Longitudinal analysis of technical efficiency of voluntary counseling and testing of HIV in China]. Beijing da xue xue bao Yi xue ban = Journal of Peking University Health sciences 41: 135-140.

65. Hyle EP, Jani IV, Lehe J, Su AE, Wood R, et al. (2014) The clinical and economic impact of point-of-care CD4 testing in mozambique and other resource-limited settings: a cost-effectiveness analysis. PLoS medicine 11: e1001725.

66. Ciaranello AL, Myer L, Kelly K, Christensen S, Daskilewicz K, et al. (2015) Point-of-care CD4 testing to inform selection of antiretroviral medications in south african antenatal clinics: a cost-effectiveness analysis. PLoS One 10: e0117751.

67. Holtgrave DR, Pinkerton SD (1997) Updates of cost of illness and quality of life estimates for use in economic evaluations of HIV prevention programs. J Acquir Immune Defic Syndr Hum Retrovirol 16: 54-62.

68. Honiden S, Sundaram V, Nease RF, Holodniy M, Lazzeroni LC, et al. ( 2006 ) The effect of diagnosis with HIV infection on health-related quality of Life. Qual Life Res 15: 69-82.

69. Schackman BR, Goldie SJ, Freedberg KA, Losina E, Brazier J, et al. (2002 ) Comparison of health state utilities using community and patient preference weights derived from a survey of patients with HIV/AIDS. Med Decis Making 22: 27-38.

70. Tengs TO, Lin TH (2002) A meta-analysis of utility estimates for HIV/AIDS. Med Decis Making 22: 475-481.

71. Long EF, Brandeau ML, Galvin CM, Vinichenko T, Tole SP, et al. (2006) Effectiveness and cost-effectiveness of strategies to expand antiretroviral therapy in St. Petersburg, Russia. AIDS 20: 2207-2215.

72. Duan Q (2014) Provincial registered drug users as many as over 160,000. Nanguo Morning Post.
